# Metabolomic changes in *Mycobacterium avium* subsp. *paratuberculosis* (MAP) challenged Holstein–Friesian cattle highlight the role of serum amino acids as indicators of immune system activation

**DOI:** 10.1007/s11306-022-01876-w

**Published:** 2022-03-23

**Authors:** Emma N. Taylor, Manfred Beckmann, Bryan K. Markey, Stephen V. Gordon, Glyn Hewinson, David Rooke, Luis A. J. Mur

**Affiliations:** 1 Aberystwyth University, Ceredigion, UK; 2 School of Veterinary Medicine, University College Dublin, Dublin, Ireland; 3 Centre of Excellence for Bovine Tuberculosis, Aberystwyth University, Ceredigion, UK; 4 ProTEM Services Ltd, West Sussex, UK

**Keywords:** *Mycobacterium avium* subspecie*s paratuberculosis*, Metabolomics, Haematology, Amino acids, Immune response

## Abstract

**Introduction:**

Paratuberculosis, commonly known as Johne’s disease, is a chronic granulomatous infection of ruminants caused by *Mycobacterium avium* subspecie*s paratuberculosis* (MAP). Clinical signs, including reduced milk yields, weight loss and diarrhoea, are typically absent until 2 to 6 years post exposure.

**Objectives:**

To identify metabolomic changes profiles of MAP challenged Holstein–Friesian (HF) cattle and correlate identified metabolites to haematological and immunological parameters.

**Methods:**

At approximately 6 weeks of age, calves (n = 9) were challenged with 3.8 × 10^9^ cells of MAP (clinical isolate CIT003) on 2 consecutive days. Additional unchallenged calves (n = 9) formed the control group. The study used biobanked serum from cattle sampled periodically from 3- to 33-months post challenge. The assessment of sera using flow infusion electrospray high resolution mass spectrometry (FIE-HRMS) for high throughput, sensitive, non-targeted metabolite fingerprinting highlighted differences in metabolite levels between the two groups.

**Results:**

In total, 25 metabolites which were differentially accumulated in MAP challenged cattle were identified, including 20 which displayed correlation to haematology parameters, particularly monocyte levels.

**Conclusion:**

The targeted metabolites suggest shifts in amino acid metabolism that could reflect immune system activation linked to MAP and as well as differences in phosphocholine levels which could reflect activation of the Th1 (tending towards pro-inflammatory) immune response. If verified by future work, selected metabolites could be used as biomarkers to diagnose and manage MAP infected cattle.

**Supplementary Information:**

The online version contains supplementary material available at 10.1007/s11306-022-01876-w.

## Introduction

Paratuberculosis, commonly known as Johne’s disease, is a chronic granulomatous infection of ruminants caused by *Mycobacterium avium* subspecies *paratuberculosis* (MAP). MAP is commonly transmitted via the ingestion of infected faeces or colostrum (Sweeney, 1996) when calves are < 6-months of age (Windsor & Whittington, 2010). Following MAP exposure, infected calves enter a prolonged incubation period before the (sub)clinical stages of disease (Koets et al., 2015). Characteristic clinical signs, such as weight loss, diarrhoea and reduced milk yields (Whitlock & Buergelt, 1996), are often absent until 2 to 6 years post infection (Salem et al., 2013).

MAP contaminated cattle demonstrate an associated annual loss of £112.9/cow (€131.8/cow), including milk yield losses of £60.6/cow (€70.7/cow) (Barratt et al., 2018). Serum ELISA data indicate that UK and Irish dairy herds have similar herd prevalence’s of 34.7% (95% CI 27.6–42.5%) (Cook et al., 2009) and 31.5% (95% CI 24.6–39.3%) (Good et al., 2009), respectively. However, these figures are likely underestimates, as serum ELISAs do not detect infected cattle in the long incubation period (Whitlock et al., 2000). Furthermore, repeat testing is needed given that the sensitivity of the serum ELISA ranges from 7 to 94% (Nielson & Toft, 2008). Interferon-γ (IFN-γ) assays can detect cell-mediated immune (CMI) responses during early infection but this response fades as infection progresses (Plain et al., 2012). Thus, diagnostic tests are currently unsuitable to identify MAP infected cattle between the initial CMI response and the onset of faecal or milk shedding in the (sub)clinical stages of infection. New tests will undoubtedly arise from an improved understanding of MAP infection and persistence mechanisms and, in this, omic approaches could prove especially useful.

As metabolites are the end products of interactions between the genome and the environment, the metabolome could be particularly informative to MAP research. Some metabolomic research on MAP has been undertaken, for example, using ^1^H nuclear magnetic resonance (^1^H NMR) spectrometry to show how MAP promotes increased energy deficits, fat metabolism and protein turnover in experimentally infected cattle (de Buck et al., 2014). Likewise, direct analysis in real time coupled with high resolution mass spectrometry (DART-HRMS) indicated similar energy and lipid-related changes in response to MAP in naturally infected and infectious cattle, in comparison to healthy controls (Tata et al., 2021).

We aimed to expand our understanding of MAP by examining the effects of MAP exposure on the metabolomic profiles of Holstein–Friesian (HF) cattle using flow infusion electrospray ionization high resolution mass spectrometry (FIE-HRMS). We also correlated the levels of targeted metabolites with cell mediated and humoral immune responses, as previously published for the same study (Britton, 2017). We demonstrate the ability of metabolomics to differentiate between MAP challenged and control cattle, as well as utilising correlation analysis with haematological and immunological parameters to suggest how the identified metabolites could reflect changes in the immune system.

## Materials and methods

### Study design, MAP culture and immunology assessments

The animal study was first described by Britton (2017) and was conducted in accordance with the Irish Health Products Regulatory Authority (Earlsfort Terrace, Dublin, Ireland) and approved by the UCD Animal Review Ethics Committee (UCD, Dublin, Ireland)**.** The study was based on 55 HF calves (54 males and 1 freemartin) sourced from 2 farms with MAP seropositivity rates of ≤ 10%. All dams were MAP faecal culture negative and seronegative. Calves were housed indoors at the Central Veterinary Research Laboratory Farm (County Kildare, Ireland) for the duration of the study. To minimise cross contamination, the two groups were housed approximately 0.5 km apart and strict biosecurity measures enforced.

Calves were randomly assigned to the MAP challenge (n = 35) or control (n = 20) group. At approximately 6-weeks of age, the challenge group received an inoculation with 3.8 × 10^9^ MAP bacterial cells (clinical isolate CIT003) on 2 consecutive days. Faecal MAP culture tests were performed at 3-, 6-, 9-, 12-, 16-, 20-, 24-, 28-, 31- and 33-months post challenge. Tissue culture was conducted upon euthanasia at either 12-, 24- or 33-months post challenge. Pathological evaluations assessed the ileum and ileocaecal valve, as well as the ileal and ileocaecal lymph nodes, for changes consistent with MAP infection. Blood samples were collected in EDTA tubes and measured for a range of haematology parameters, including monocytes (% and × 10^9^/L) (Supplementary Table 1).

The CMI response was assessed 2-, 3-, 6-, 10-, 12-, 16-, 20-, 24-, 28-, 31- and 33-months post challenge using the Bovigam IFNɣ release assay (IGRA) (Prionics AG, Schlieren-Zurich, Switzerland) following manufacturer’s instructions. IGRA results were determined by subtracting the mean delta optical density (∆OD) of the phosphate buffered saline (PBS) stimulated wells from antigen stimulated wells. In line with IDEXX-criteria (Jungersen et al., 2022), results were classified as MAP-positive if the PPDa stimulated wells exhibited an ∆OD 0.999 higher than the PBS stimulated wells and if the PPDb stimulated wells divided by the mean of the PPDa wells was below 0.71.

The humoral immune response was assessed pre-challenge, 3-, 6-, 10-, 12-, 15-, 16-, 20-, 21-, 24-, 27-, 28-, 30-, 31- and 33-months post challenge using the Johne’s Identification and Verification ELISA (IDEXX laboratories Inc., Maine, USA) following manufacturer’s instructions. Initially, samples were tested using the Johne’s Identification ELISA, samples with a positive control ratio (S/P%) < 45% were classified as MAP negative. All samples ≥ 45% were retested with the Johne’s Verification ELISA. S/P% values between 45–54% were deemed inconclusive and S/P% values ≥ 55% were classified as MAP positive.

### Untargeted metabolite fingerprinting by flow infusion electrospray ionization high resolution mass spectrometry (FIE-HRMS)

Sera from 9 MAP challenged and 9 control cows, which were euthanized 33-months post MAP challenge and sampled 3-, 6-, 9-, 12-, 15-, 21-, 24-, 28-, 31- and 33-months post MAP challenge were prepared as described by Beckman et al., (2008) with minor amendments. Samples were defrosted on ice, vortexed for 5 s and 200 µL was pipetted into 1520 µL pre-chilled solvent mix (methanol/chloroform [4/1]) containing 1 micro-spoon of glass beads. Samples were then vortexed for 5 s, shaken for 15 min at + 4 °C and kept at − 20 °C for 20 min. Following centrifugation at 22,000×*g* and 4 °C for 5 min, 100 µL of the serum supernatant was transferred into mass spectrometry vials along with 100 µL methanol/water [70/30]. For each sample, 20 µL were injected into a flow of 60 µL per minute water–methanol, at a ratio of 70% water and 30% methanol, using a Surveyor flow system into a Q Exactive plus mass analyser instrument with UHPLC system (Thermo Fisher Scientific©, Bremen, Germany) for high throughput FIE-HRMS. Data acquisition for each serum sample was done by alternating the positive and negative ionisation modes, throughout four different scan ranges (15–110 m*/z*, 100–220 m*/z*, 210–510 m*/z*, 500–1200 m*/z*) with an acquisition time of 2 min. Each serum sample was analysed in each scan range once. The derived data matrices are available at MetaboLights (MTBLS2963).

### Statistical analysis

Metabolomic data were analysed using MetaboAnalyst 4 (Chong et al., 2019). Data were subjected to interquartile range-based filtering, log_10_ transformations and Pareto scaling. Time series analyses used false discovery rate (FDR) adjusted two-way ANOVA tests to identify *m/z* which significantly (p values < 0.05) differed between experimental classes. Partial least squares—discriminate analysis (PLS-DA) and variables of importance for the projection (VIP) scores (> 1) visualised the differences between the experimental classes. Major sources of variation were displayed using unsupervised hierarchical clustering analysis (HCA). Area under the curve (AUC) assessments based on sensitivity and specificity estimates was used to determine the accuracy of the target *m/z* as potential biomarkers. Random Forest (RF) was used as an alternative multivariate classification test. This uses a confusion matrix to estimate how often a given *m/z* variable would give an estimate of the classification error.

Significant *m/z* were identified based on accurate mass (5 ppm resolution) using the mummichog algorithm. All isotopes/adducts were considered. Metabolite set enrichment analysis (MSEA) using over representation analysis (ORA) was used to highlight key pathways and biological patterns. Correlation analysis between identified metabolites, immunology biomarkers and haematology parameters were performed using Pearson’s correlation coefficient.

## Results

### The MAP challenge study

The MAP challenge study has already been described by Britton (2017) and details are here included to aid comprehension of these follow-on metabolomic experiments. All cattle were faecal and tissue culture MAP-negative throughout the study. Post-euthanasia examinations of MAP challenged cattle highlighted a number of gross pathological changes, such as thickening of the ileum and enlarged ileocaecal lymph nodes. However, all Ziehl–Neelsen (ZN) stained tissue smears were negative. MAP challenged cattle showed significantly higher IGRA (∆OD) expression in response to purified protein derivative (PPD) from *M. avium* subsp. *avium* (PPDa) or *M. avium* subsp. *paratuberculosis* (PPDj) stimulation at 2-, 6-, 10-, 12-, 16-, 28-, 31- and 33- months post MAP challenge (Mann Whitney test; p < 0.05) compared to controls. Likewise, purified protein derivative from *M. bovis* PPD (PPDb) stimulation led to significantly higher IGRA (∆OD) expression in infected cattle at 6-, 10-, 12-, 16-, 28-, 31- and 33- months post MAP challenge (Mann Whitney test; p < 0.05) compared to controls. Over the duration of the study, 7 MAP challenged and 2 control cattle were ELISA positive and a further 3 MAP challenged cattle displayed inconclusive results. Analysis of haematology parameters, including lymphocytes and monocytes, at 12-, 24- and 33-months post MAP challenge demonstrated no significant results (p > 0.05) (Supplementary Table 1).

### Metabolomic changes

**Fig. 1:**
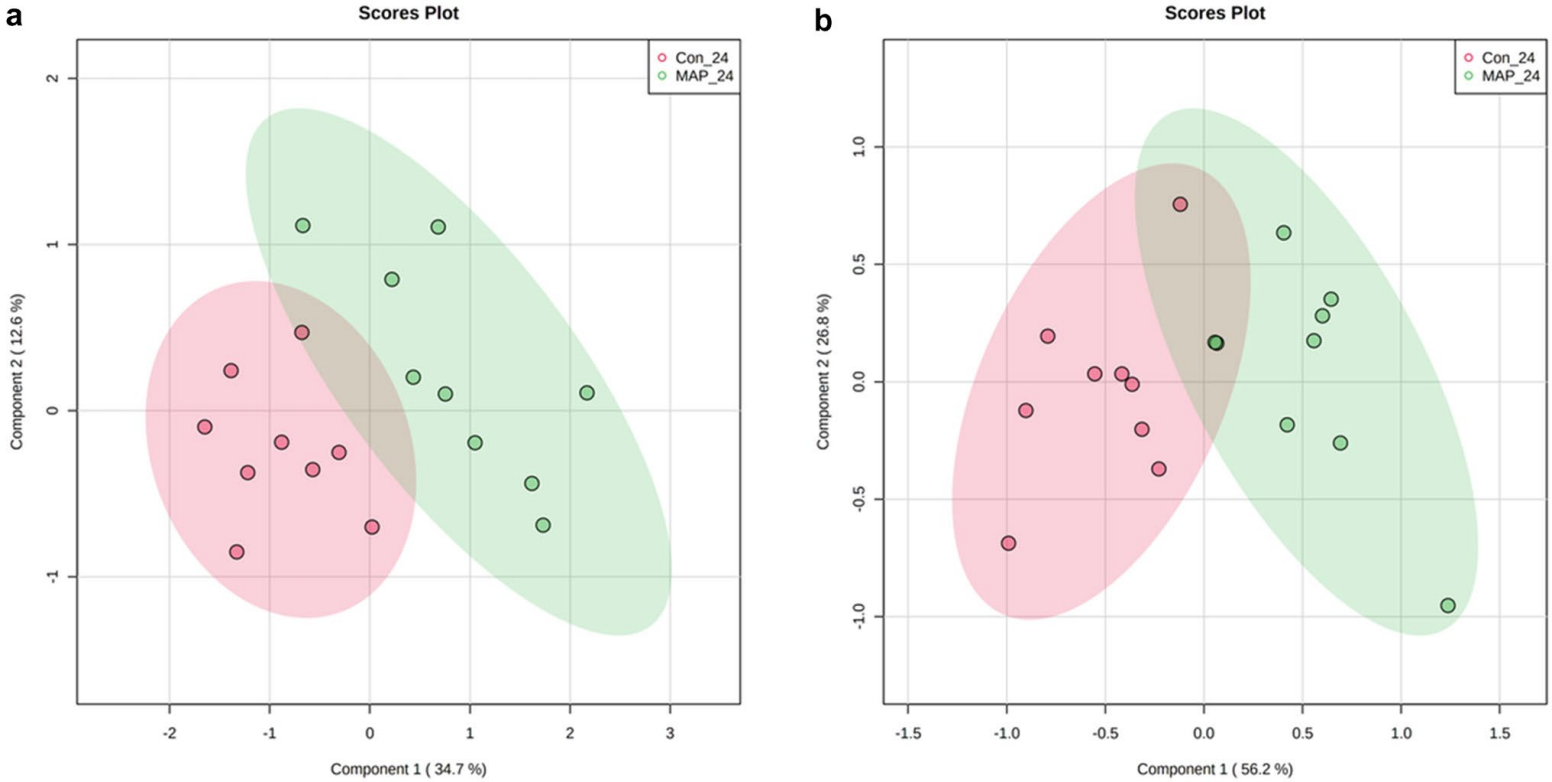
Partial least-squares discriminant analysis (PLS-DA) for MAP challenged and control cattle in the **a** negative ionization and **b** positive ionization modes 24-months post MAP-challenge. The light red and green ellipses represent 95% confidence intervals

PLS-DA of the serum metabolite profiles derived from both negative and positive ionisation modes *m/z* exhibited some discrimination between MAP challenged and control cattle, albeit with overlapping of 95% confidence intervals, PLS-DA models 24-months post MAP inoculation are shown in Fig. 1. The sources of variation are displayed based on their VIP scores from PLS-DA models and were classified based on differences between control and MAP inoculated datasets. Supplementary Figs. 1 and 2 display the VIP scores (> 1) for the PLS-DA models 24-months post MAP inoculation. Nevertheless, the sources of variation between the experimental classes were identified based on time series two-way ANOVA (p < 0.05) and corrected for FDR. These assessments identified 25 significant metabolites (Table 1). Discrete metabolites showed either relative increases or decreases in MAP challenged samples compared to controls (Supplementary Figs. 3 and 4). Table 1 indicates that identified metabolites belonged to various (sub) classes, although. there is a notable accumulation of 6 metabolites from the amino acids, peptides and analogues subclass. MSEA using ORA highlighted the effect of MAP challenge on phosphatidylcholine biosynthesis and alanine metabolism in negative ionisation mode (p < 0.05) (Supplementary Fig. 5) and arginine and proline metabolism in positive ionisation mode (p < 0.05) (Supplementary Fig. 6).

**Table 1:** Metabolites differentiating between MAP challenged and control cattle

Class	Subclass	Metabolite	Ionization mode	*p* values		
				MAP challenge	Time	Interaction
Benzene and substituted derivatives	Benzoic acids and derivatives	Hippuric acid	Negative	3.74 × 10^–2^	2.12 × 10^–18^	5.54 × 10^–3^
Biotin and derivatives	–	Biotin	Negative	1.85 × 10^–3^	4.72 × 10^–6^	7.83 × 10^–3^
Carboxylic acids and derivatives	Amino acids, peptides, and analogues	(S)-Ureidoglycolic acid	Positive	1.09 × 10^–2^	5.17 × 10^–16^	3.06 × 10^–1^
		3-Sulfinoalanine	Positive	4.18 × 10^–2^	3.67 × 10^–2^	9.39 × 10^–2^
		Citrulline	Positive	1.85 × 10^–2^	3.51 × 10^–4^	5.10 × 10^–2^
		Methionine	Negative	4.74 × 10^–2^	1.36 × 10^–4^	5.35 × 10^–1^
		Proline	Positive	3.61 × 10^–2^	1.25 × 10^–17^	8.34 × 10^–3^
		Phenylacetylglycine	Negative	2.85 × 10^–2^	1.57 × 10^–11^	1.19 × 10^–6^
	Carboxylic acids	Glyoxylic acid	Negative	2.66 × 10^–2^	1.31 × 10^–3^	2.74 × 10^–1^
Diazines	Pyrimidines and pyrimidine derivatives	6-Thioxanthine 5'-monophosphate	Negative	3.68 × 10^–3^	2.00 × 10^–17^	1.56 × 10^–7^
Fatty acyls	Fatty acids and conjugates	Arachidic acid	Negative	3.02 × 10^–2^	2.31 × 10^–15^	6.43 × 10^–1^
Glycerolipids	Glycosylglycerols	Galactosylglycerol	Positive	1.55 × 10^–2^	6.80 × 10^–12^	4.88 × 10^–3^
Keto acids and derivatives	Medium-chain keto acids and derivatives	Maleylacetoacetic acid	Negative	3.09 × 10^–3^	2.48 × 10^–8^	4.78 × 10^–6^
	Short-chain keto acids and derivatives	2-Oxosuccinamate	Negative	1.55 × 10^–2^	4.75 × 10^–6^	1.50 × 10^–1^
Organic phosphoric acids and derivatives	Phosphate esters	O-Phosphoethanolamine	Negative	4.89 × 10^–2^	1.10 × 10^–6^	6.89 × 10^–2^
Organonitrogen compounds	Quaternary ammonium salts	Phosphorylcholine	Negative	2.32 × 10^–2^	2.52 × 10^–13^	4.88 × 10^–2^
	Carbohydrates and carbohydrate conjugates	Xylose	Positive	3.61 × 10^–2^	3.10 × 10^–7^	4.27 × 10^–1^
		N-Acetylneuraminate	Negative	1.19 × 10^–2^	4.61 × 10^–4^	9.37 × 10^–1^
	Carbonyl compounds	4-Aminobutyraldehyde	Negative	3.68 × 10^–2^	1.43 × 10^–3^	2.89 × 10^–1^
		Acetaldehyde	Negative	4.94 × 10^–4^	3.29 × 10^–12^	3.73 × 10^–5^
		Malonyl-CoA	Positive	3.16 × 10^–2^	7.04 × 10^–18^	6.44 × 10^–1^
Purine nucleosides	Purine 2'-deoxyribonucleosides	Deoxyadenosine	Negative	2.32 × 10^–2^	2.27 × 10^–3^	3.29 × 10^–2^
Pyrimidine nucleosides	–	Cytidine	Negative	5.89 × 10^–3^	4.49 × 10^–4^	3.51 × 10^–3^
		Uridine	Negative	4.89 × 10^–2^	1.72 × 10^–4^	1.00 × 10^–1^
Steroids and steroid derivatives	Bile acids, alcohols and derivatives	3a,7a,12a-Trihydroxy-5b-cholestanoic acid	Positive	3.61 × 10^–2^	9.05 × 10^–7^	4.37 × 10^–3^

**Fig. 2:**
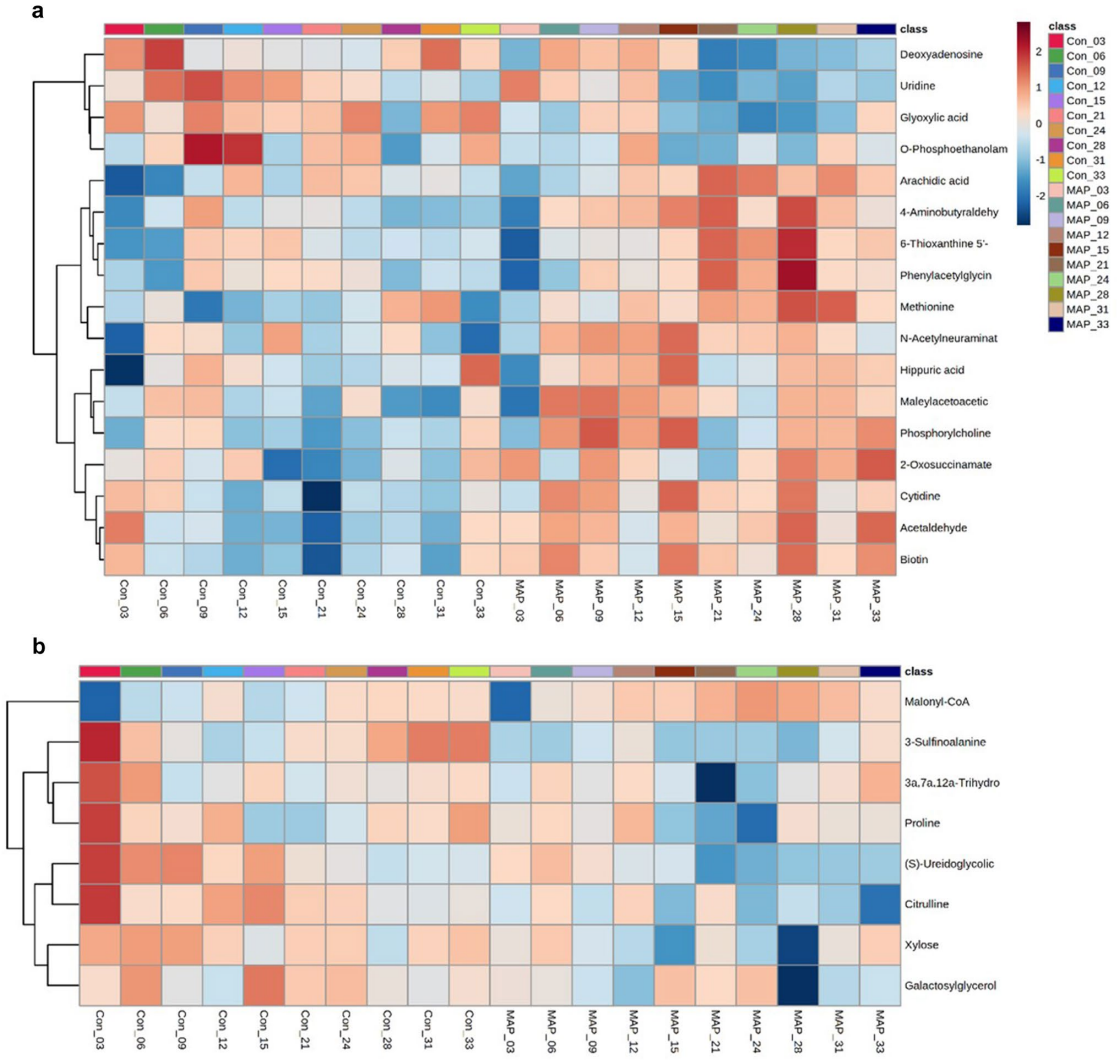
Major metabolite changes differentiating between MAP challenged and control cattle between $\sim$ 3-month and 33-months post MAP inoculation, Metabolites detected in **a** negative and **b** positive ionization modes

Of the 25 metabolites identified, all were significantly affected by time and 12 out of 25 metabolites by MAP challenge status *time (p < 0.05) (Table 1). Therefore, the levels of the 25 metabolites derived from either negative (Fig. 2a) and positive ionisation modes (Fig. 2b) are displayed over time using heat maps. Considerable variation in *m/z* was observed, particularly at early time points but there appeared to be improved consistency from 15-months post MAP challenge. Within the negative ionisation data, the abundance of metabolites involved in nucleotide metabolism (deoxyadenosine and uridine), the Krebs cycle (glyoxylic acid) and phosphatidylcholine biosynthesis (*o*-phosphoethanolamine) decreased within MAP challenged cattle. However, the majority of metabolite changes showed increases, particularly two keto acids (maleylacetoacetic acid and 2-oxosuccinamate). In contrast, in the positive ionisation data, levels of all metabolites decreased in MAP challenged cattle, except for malonyl-CoA. Interestingly, 4 out of 7 metabolites that showed a decrease belong to the amino acids, peptides and analogues subclass.

The specificities and sensitivities of each of the targeted metabolites were assessed using AUC calculations (Supplementary Table 2). Of the 25 identified metabolites, 5 metabolites (2-oxosuccinamate, 6-thioxanthine 5'-monophosphate, acetaldehyde, biotin and (S)-ureidoglycolate) showed AUC values of > 0.75 from 24-months post challenge. Other metabolites detected using the positive ionisation mode (3a,7a,12a-Trihydroxy-5b-cholestanoic acid, proline and (S)-ureidoglycolate) exhibited discriminatory AUC values of > 0.90 within 3-months post challenge. However, these AUC values dramatically decreased to < 0.60 within 6-months post challenge. Thus, AUC calculations suggested that no metabolites were able to distinguish between experimental classes at all stages of the study. These results were consistent with the trends shown in Fig. 2a and 2b. Additionally, RF analysis reported class errors of 0.00 for both MAP challenge and control cattle 33-months post challenge, respectively.

**Fig. 3:**
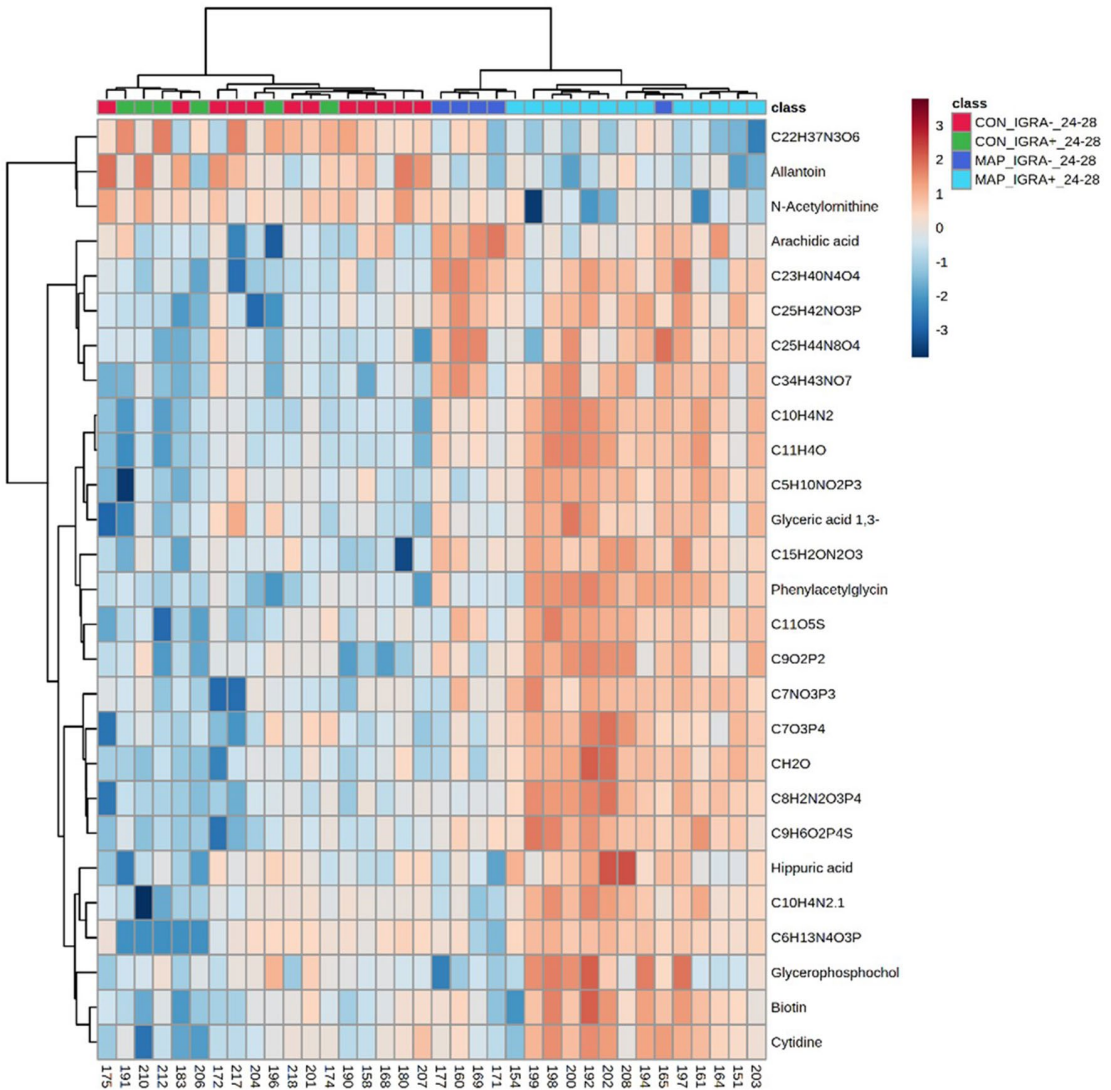
Significant metabolite changes (negative ionization model) differentiating between MAP challenge status and interferon-y release assay IDEXX criteria MAP interpretation results between 24 and 28-months post MAP challenge

We next assessed the ability of metabolomes to differentiate between MAP challenge status and IGRA status. Significant metabolite changes that differentiate between the experimental classes are displayed using a heat map many of which could not be unambiguously identified, and their molecular formulae are provided (Fig. 3). There was limited overlap in the metabolites identified based on MAP or IGRA status with only phenylacetylglycine, hippuric acid, biotin and cytidine being common to both analyses. The heat map showed that it was possible to differentiate between MAP challenged and control cattle but not between control cattle which were IGRA positive or negative. These findings were reinforced by Pearson’s correlation analysis which showed poor correlation between identified metabolites and responses to PPDa, PPDb and PPDj stimulation (Supplementary Fig. 7). It should be noted that Britton (2017) showed significant differences between IGRA expression in response to PPDa and PPDj 2-, 6-, 10-, 12-, 16-, 28-, 31- and 33-months post MAP challenge.

**Fig. 4:**
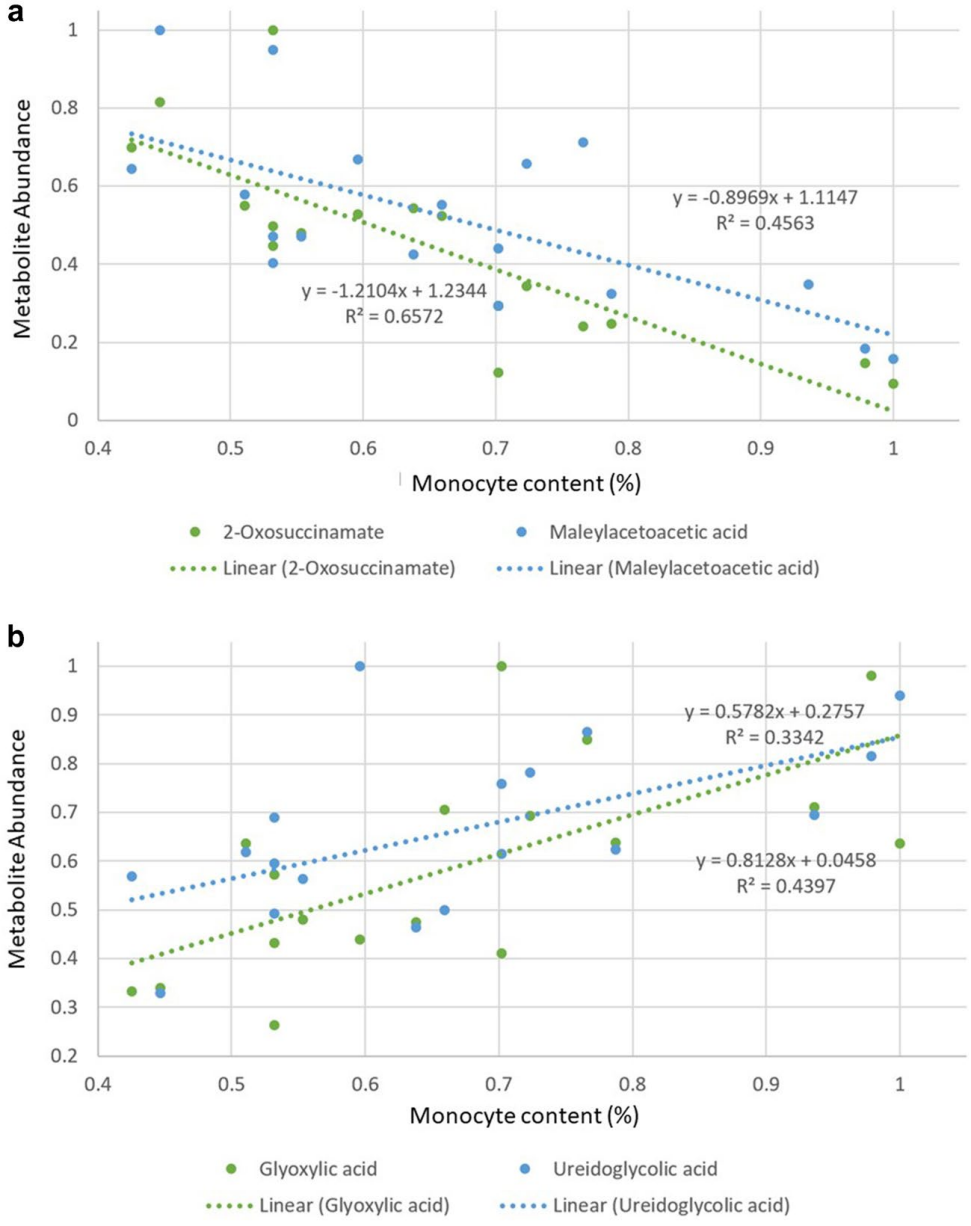
Relationship between monocytes content (%) and **a** 2-oxosuccinamate and maleylacetoacetic acid as well as **b** glyoxylic acid and ureidoglycolic 33-months post MAP challenge

Pearson’s correlation analyses were next used to examine the relationship between 25 identified metabolites, the CMI and humoral immune responses, as well as the 17 haematological parameters examined. Of the 525 analysed correlations, 28 were positively correlated (correlation co-efficient > 0.4) and 26 were negatively correlated (correlation co-efficient < -0.4). Of the 54 observed correlations, 53 were between identified metabolites and haematological parameters (Supplementary Fig. 8). As also shown by Britton (2017), no significant differences were observed within the haematology parameters of MAP challenged and control cattle. Nevertheless, most of the haematology parameters showed significant correlations with 1 or more identified metabolites. Monocytes (%) and MCH (pg) were most frequently correlated to metabolites, displaying 11 and 6 correlations, respectively, (Supplementary Table 3). Keto acids (2-oxosuccinamate and maleylacetoacetic acid) showed the most significant negative correlation (Supplementary Fig. 7 and Fig. 4a), whilst glyoxylic acid and (S)*-*ureidoglycolic acid from the carboxylic acids and derivatives class displayed the strongest positive correlation to monocytes (Supplementary Fig. 9 and Fig. 4b).

**Fig. 5:**
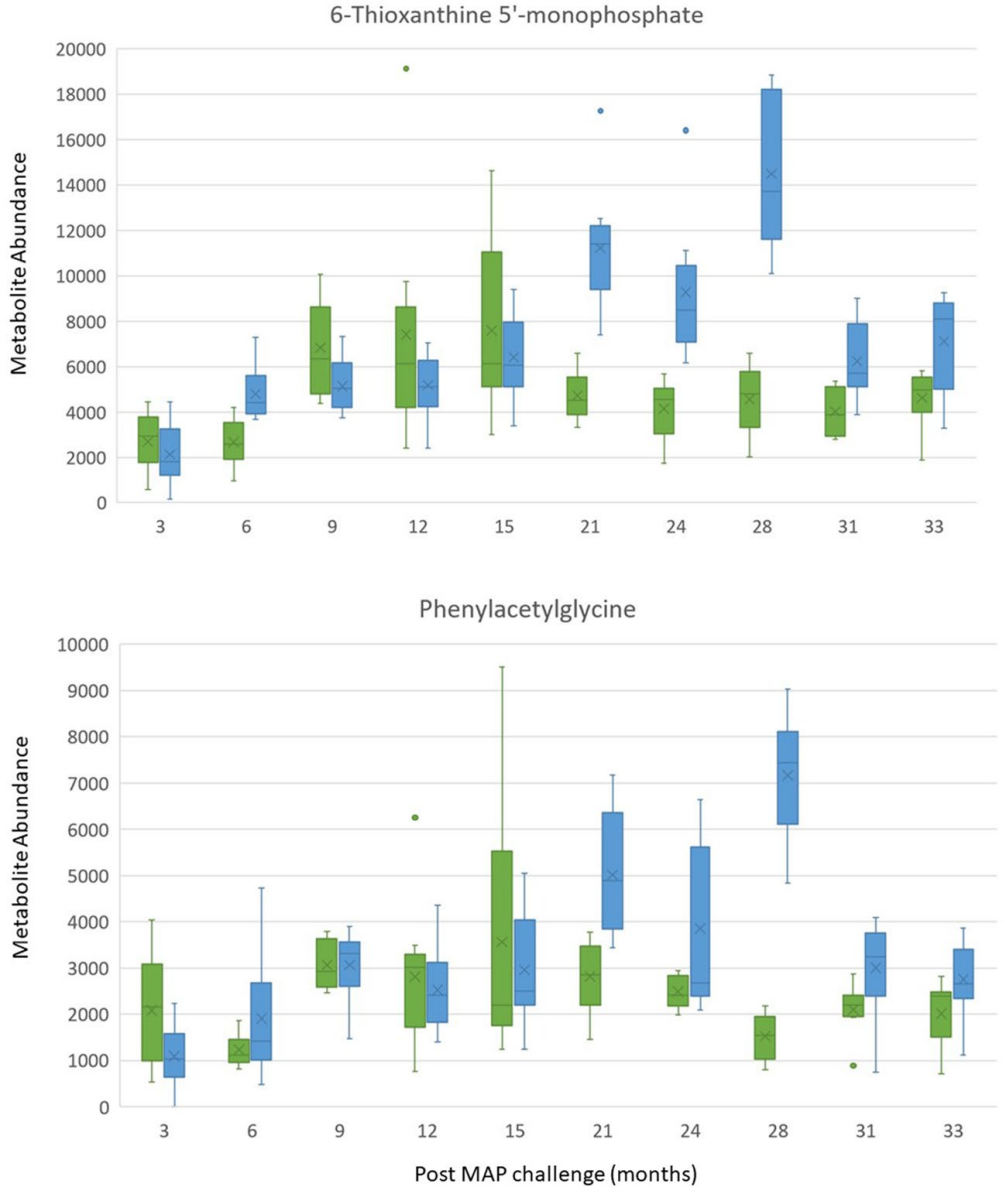
Box and whisker plots of metabolites which display reduced overlapping between groups, MAP challenged and control cattle, from 21 months post MAP challenge. Blue boxplpots = MAP challenged cattle, green boxplots = control cattle (Color figure online)

Blood from MAP challenged cattle stimulated with PPDa exhibited positive correlation with phosphocholine, but blood stimulated with PPDb or PPDj, or serum ELISA data showed either no, or only a weak correlation with identified metabolites (Supplementary Fig. 7). Interestingly, 6-thioxanthine 5'-monophosphate and phenylacetylglycine exhibited peaks 28-months post MAP-challenge (Fig. 5a and 5b). Despite showing a weak overall correlation to PPDa, PPDb and PPDj, this peak coincides with the 2^nd^ peak in IGRA (∆OD) expression in response to PPDa, PPDb and PPDj stimulation (Supplementary Fig. 10).

## Discussion

Paratuberculosis is a chronic disease that represents an on-going challenge to farmers. The management of MAP will be improved if the disease is better understood and, in this, the application of ‘omic approaches have a major role to play. However, metabolomics has only rarely been applied in MAP research. This stated, metabolomic approaches have been used to show the impact of MAP on energy, lipid and protein metabolism within naturally and experimentally infected cattle (De Buck et al., 2014; Tata et al., 2021). We sought to extend such studies by examining the effects of experimentally challenging cattle with MAP with a view to providing new clinical insights and possibly identifying novel metabolite biomarkers. To provide additional insights and to provide wider context, we sought to correlate the key, identified metabolites with cell mediated and humoral immune responses, as well as haematological parameters. FIE-HRMS does not utilise prior chromatographic separation and this allows a wide dynamic range of metabolite content to be measured with a high sensitivity. This, coupled with a high resolution (3 ppm) that facilitates metabolite identification, allows the chemical content of a sample to be more comprehensively profiled (Draper et al., 2013). The value of this approach was demonstrated when FIE-HRMS successfully discriminated between MAP challenged and control cattle 24-months post challenge (Fig. 1) despite a small sample size. This was an important observation as faecal and tissues cultures were unable to confirm the presence of MAP within MAP challenged cattle, despite a number of gross pathological changes, such as thickening of the ileum and enlarged ileocaecal lymph nodes.This stated, other tests results could be linked with MAP infection, including IGRA expression in response to PPDa, PPDb and PPDj stimulation being significantly higher in MAP challenged cattle.

Time series two-way ANOVA (p < 0.05) analyses corrected for FDR indicated how identified metabolites significantly changed over the duration of the study (Table 1). However, within the experimental classes, as shown by HCA, considerable variation was observed at the early time points, but this reduced from 15-months post MAP challenge (Fig. 2). Considering that male bovine puberty occurs between 37 and 50 weeks of age (Rawlings et al., 2008), sexual maturity is unlikely to be a major driver of metabolite fluctuations in this experiment. However, HF steers continue to grow until a liveweight of ~ 620 kg is achieved at approximately 24 months of age (O’Riordan et al., 2019). Additionally, the impact of diet on the bovine metabolome is well-documented (Yang et al., 2018). Therefore, a mixture of growth and dietary factors are likely to contribute to metabolite variation up to 15-months post MAP challenge. Similarly, a longitudinal trial conducted by De Buck et al., (2014) of MAP infected HF heifers and age-matched controls sampled over 17-months noted that key variables were significantly affected by time. These metabolomic changes were suggested to reflect development and dietary changes.

### MAP exposure is typified by amino acid and phospholipid changes in the serum metabolome.

Considering the discriminatory metabolites, it was notable that amino acids, peptides, and analogues subclass (class—carboxylic acids and derivatives) showed prominent changes following MAP challenge. Methionine and phenylacetylglycine levels increased, whilst (S)-ureidoglycolic acid, 3-sulfinoalanine, citrulline and proline decreased (Fig. 2). Although these metabolites have not been previously associated to MAP, the effect of MAP on amino acid levels is well-documented. Thus, De Buck et al., (2014) reported significant increases in amino acids; tyrosine, threonine, isoleucine, leucine and asparagine, within MAP infected cattle. Likewise, Tata et al., (2021) found increased creatine, creatinine and tryptamine levels within MAP infected and infectious cattle, compared to controls. These changes were suggested to be linked to a reduced absorptive capacity due to MAP induced inflammation (De Buck et al., 2014). However, this does not accord with our observations as only 3 MAP challenged cattle included in the metabolomic analysis showed thickening and corrugation of the ileum, and none exhibited positive ZN stains (Britton, 2017). It is may be that a reduced absorptive capacity is not the cause of altered amino acid metabolism.

Other amino acid changes could be more easily associated with the possible effects of MAP infection. MSEA using ORA showed that alanine metabolism was significantly affected by MAP challenge (Supplementary Fig. 5). Biotin was significantly increased but glyoxylic acid was significantly decreased in MAP challenged cattle (Fig. 2a). These results suggest that alanine was converted to pyruvic acid by NAD(H) dependent L-alanine dehydrogenase (ALD), in conjunction with glyoxylic acid being converted to glycine by glycine dehydrogenase (Giffin et al., 2011). Pyruvic acid would then be converted to oxalacetic acid, via pyruvate carboxylase (using biotin as a co-factor), to feed the citric acid cycle (SMPDB, 2019). Previous studies have demonstrated the oxidative deamination of alanine to pyruvate to aid the growth of *Mycobacterium tuberculosis* (Giffin et al., 2011), *Mycobacterium bovis* Bacillus Calmette-Guérin (*M. bovis* BCG) (Chen et al., 2003) and *Mycobacterium smegmatis* (Feng et al., 2002). Therefore, these changes in host sera maybe indicative of MAP metabolism within challenged cattle.

MSEA using ORA also suggests that phosphatidylcholine biosynthesis was significantly affected by MAP challenge (Supplementary Fig. S5). This indicated that *o*-phosphoethanolamine and N-trimethyl-2-aminoethylphosphonate (also known as phosphorylcholine) differed following MAP inoculation. Reduced levels of phosphocholine-containing lipids, such as phosphatidylcholines, choline plasmalogens, and sphingomyelins have previously been identified as features of MAP infected cattle that were displaying clinical signs (Wood et al., 2018). Our data suggests that MAP challenges induce minor changes in phosphatidylcholine biosynthesis within MAP challenged cattle during the incubation period, but major shifts do not develop until the clinical stage of infection. Furthermore, our correlation analyses indicated a positive correlation between PPDa stimulated blood and phosphorylcholine. Both PPDa stimulated blood and phosphorylcholine showed a two-peaked pattern, starting out at a high level but reducing 21 to 24-months post MAP challenge before increasing again. (Fig. 2a; Supplementary Fig. 10). PPDa antigens highlight Th1 immune responses (mostly pro-inflammatory) and IFN-γ has been shown to increase phosphatidylcholine hydrolysis and phosphorylcholine production in human HeLa-S3 cells (Pfeffer et al., 1990). Thus, elevated phosphorylcholine expression is suggestive of Th1 immune response activation.

### The MAP-challenged cattle metabolome may reflect immunological changes

To develop our understanding of the biological relevance of the metabolomic differences following MAP infection, we correlated these with key haematological data as provided by Britton (2017). This indicated correlations (− 0.4 < correlation co-efficient > 0.4) between 53 of the metabolites and haematology parameters, particularly MCH and monocytes (Supplementary Table 3; Supplementary Fig. 8). At the onset of infection, monocytes are recruited to the affected tissue where they differentiate into macrophages or dendritic cells (Yang et al., 2014). Macrophages play important roles in destroying MAP-infected tissues and therefore preventing disease progression (Jenvey et al., 2019). Interestingly, alanine metabolism, which was significantly affected by MAP challenge (Supplementary Fig. 5), is altered in M2 macrophages (Abuawad et al., 2020) and the alanine-associated metabolite, glyoxylic acid was correlated with monocytes levels (Supplementary Fig. 8). Th1 cells secrete cytokines such as IFN-γ, to promote macrophage activation and cytotoxic T cell proliferation and MAP elicited changes within the alanine metabolism are likely to contribute to this immune response through M2 macrophage recruitment. Beyond alanine metabolism, monocytes displayed the strongest correlation with metabolites from the keto acids and derivatives class; 2-oxosuccinamate and maleylacetoacetic acid (Fig. 4a; Supplementary Fig. 8) which are, respectively, derivatives of asparagine and tyrosine (Matthews, 2007; Quash et al., 2003). Significantly, we also found that tyrosine and asparagine were significantly different in MAP challenged cattle (Table 1) and exhibited high AUC values, especially in the latter phase of the experimental time course (Supplementary Table 2). These data suggest that MAP-elicited amino acid processing arises from immunological responses to infection and therefore, may have utility as biomarkers.

Differences in N-acetylneuraminate (the predominant sialic acid) levels reinforce these findings. *M. tuberculosis* infected patients demonstrate positive correlation between sialic acid and mycobacterial load (Xia et al., 2020). Moreover, *M. tuberculosis*, alongside other bacteria, exhibit sialic acid decorated proteins which act as receptors and are capable of masking recognition sites and provide protection from humoral and cellular immune system responses (Bhagavat & Chandra, 2013). Therefore, elevated levels of N-acetylneuraminate within MAP challenged cattle (Fig. 2a) may reflect bacterial load and be indicative of MAP using altering sialic acid patterns on proteins to evade the immune system.

## Conclusion

Metabolomic analysis of MAP challenged and control cattle between 3- and 33-months post MAP challenge using untargeted FIE-MS highlighted metabolites indicative of MAP exposure. In total, 25 metabolites which were differentially accumulated in MAP challenged cattle were identified, including 20 which displayed significant correlation to haematological parameters. The targeted metabolites suggest that MAP induces changes in amino acid and phosphocholine levels which are suggestive of a Th1 immune response. Future work could include identifying these metabolites in naturally MAP infected cattle and tissue or faecal culture positive cattle, as well as measuring their accumulations in *M. bovis* infected cattle to verify their specificity to MAP.

## Supplementary Information

Below is the link to the electronic supplementary material.

Supplementary file1 (DOCX 5430 kb)

## Data Availability

The metabolomics matrices are available at MetaboLights (MTBLS2963) and metadata reported in this paper are available in the supplementary data.

## Code availability

The database reported in this study is accessible via https://www.metaboanalyst.ca/
