# Supplementary material for: Metabolomic changes in Mycobacterium avium subsp. paratuberculosis (MAP) challenged Holstein–Friesian cattle highlight the role of serum amino acids as indicators of immune system activation
Source: Metabolomics. 2022 Mar 23;18(4):21. doi: 10.1007/s11306-022-01876-w (PMC8942901; doi:10.1007/s11306-022-01876-w)
Supplement: Supplementary file 1 — Supplementary file1 (DOCX 5430 kb) [file 11306_2022_1876_MOESM1_ESM.docx]

**Supplementary Figures and Tables**

**
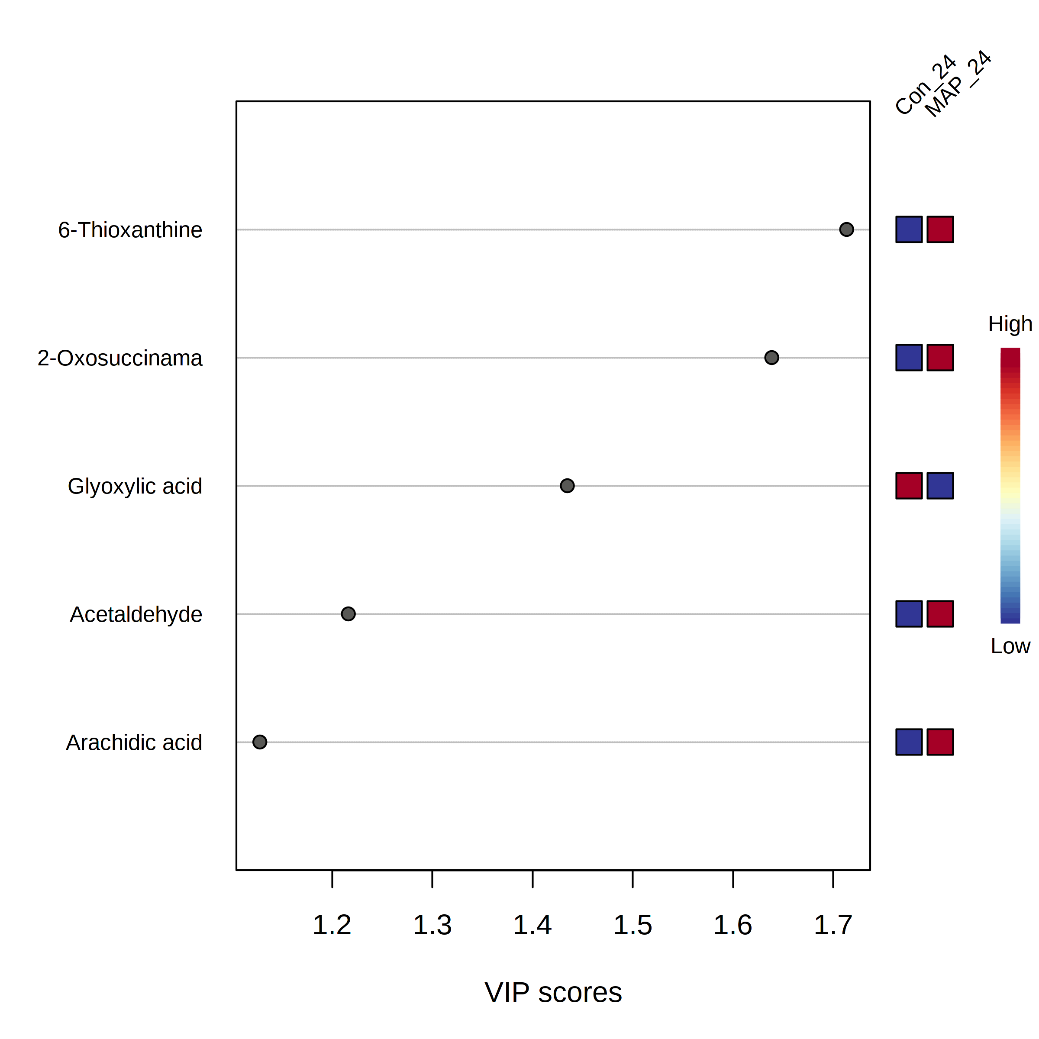
**

**Supplementary Fig 1** Variable Importance in Projection (VIP) score plots (>1) produced by partial least squares - discriminate analysis (PLS-DA) of metabolites differentially expressed in MAP challenged and control cattle in the negative ionization mode 24-months post MAP-challenge.


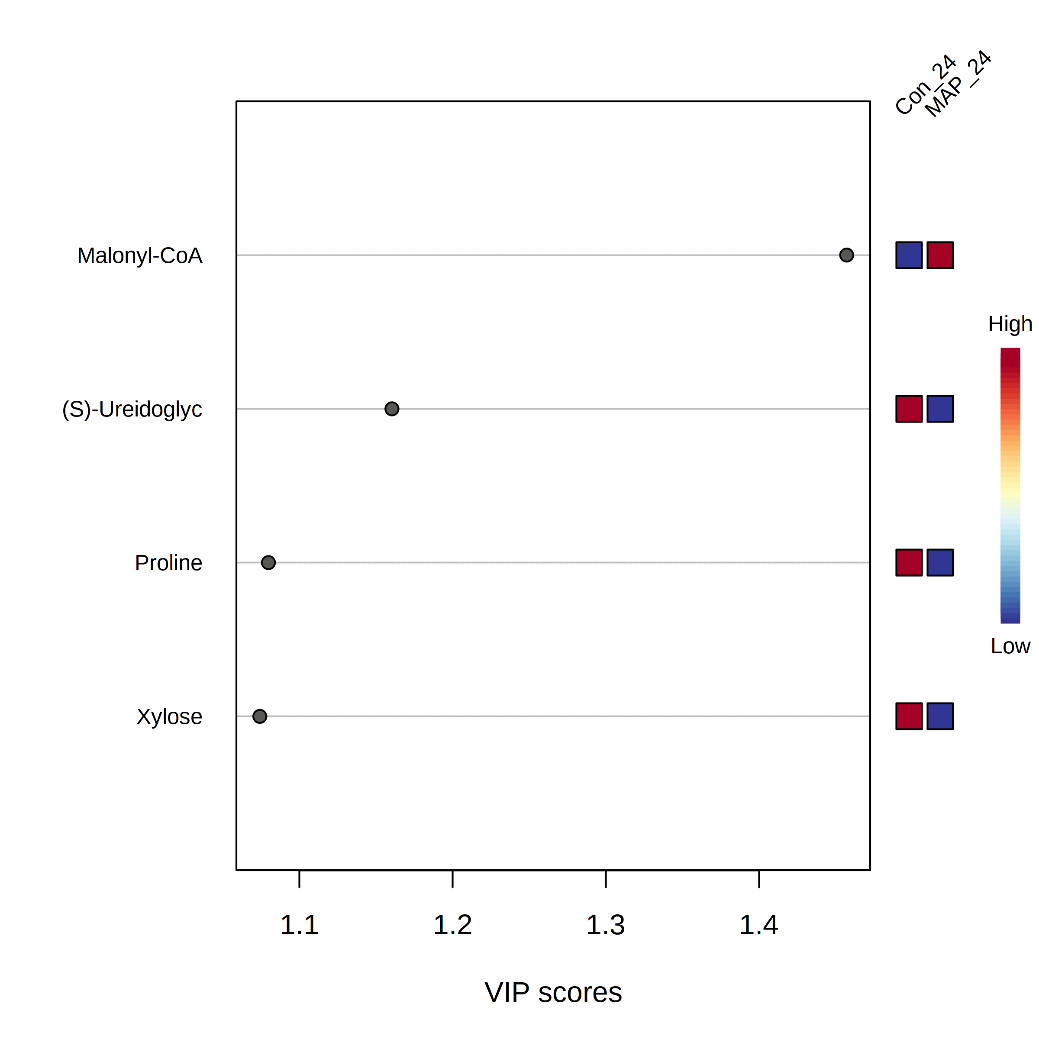


**Supplementary Fig 2** Variable Importance in Projection (VIP) score plots (>1) produced by partial least squares - discriminate analysis (PLS-DA) of metabolites differentially expressed in MAP challenged and control cattle in the positive ionization mode 24-months post MAP-challenge.


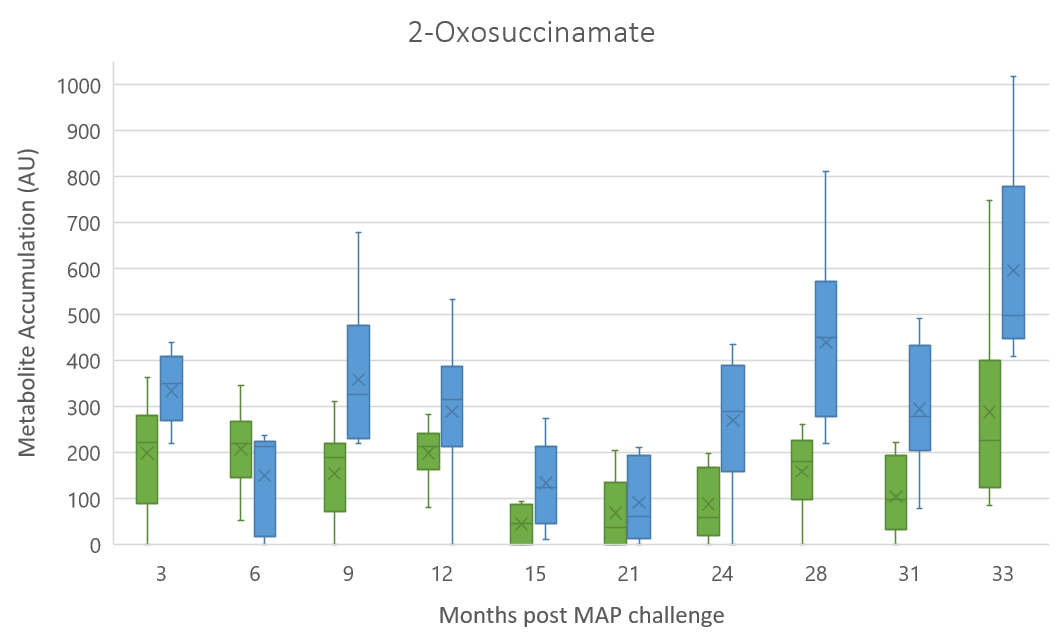

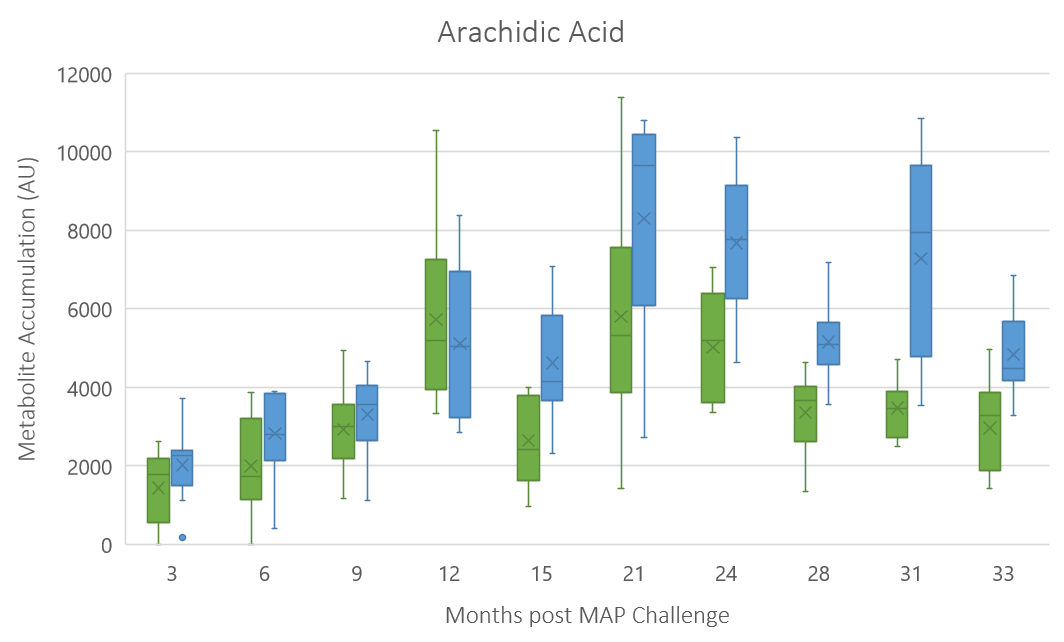

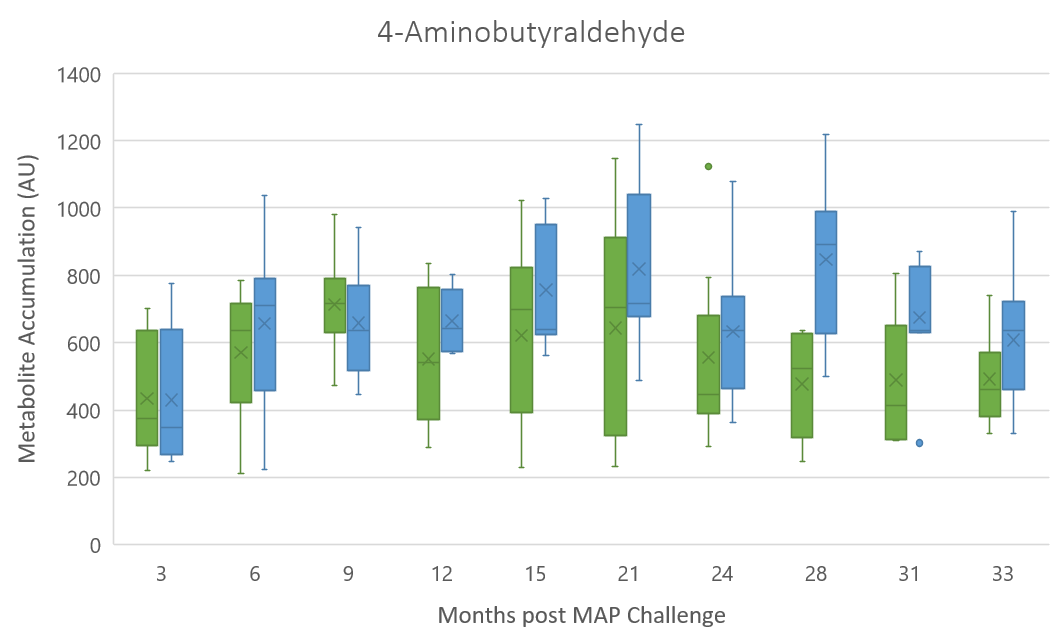

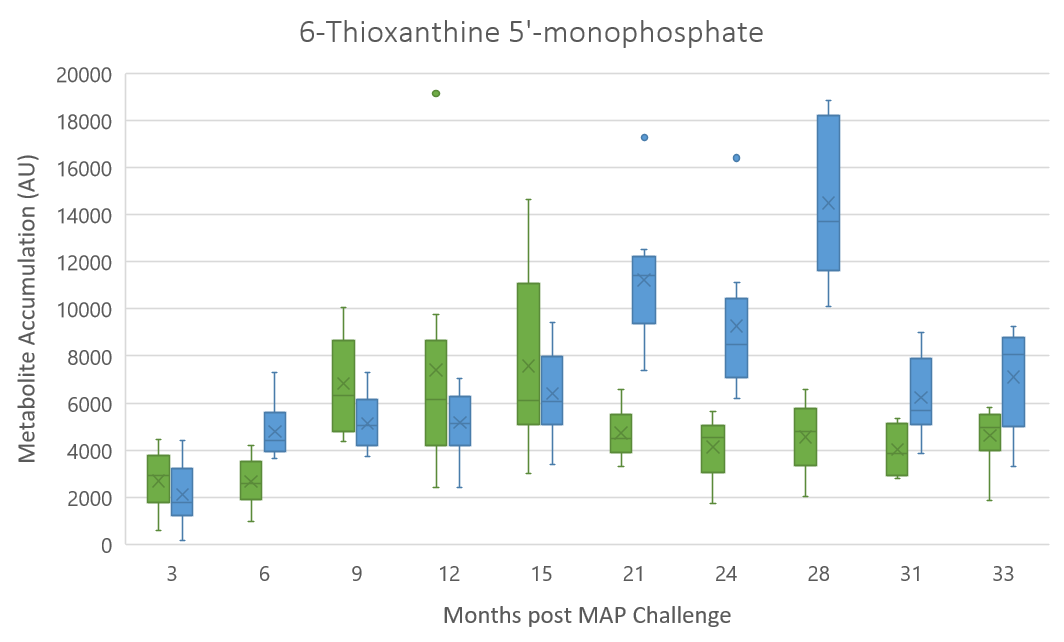

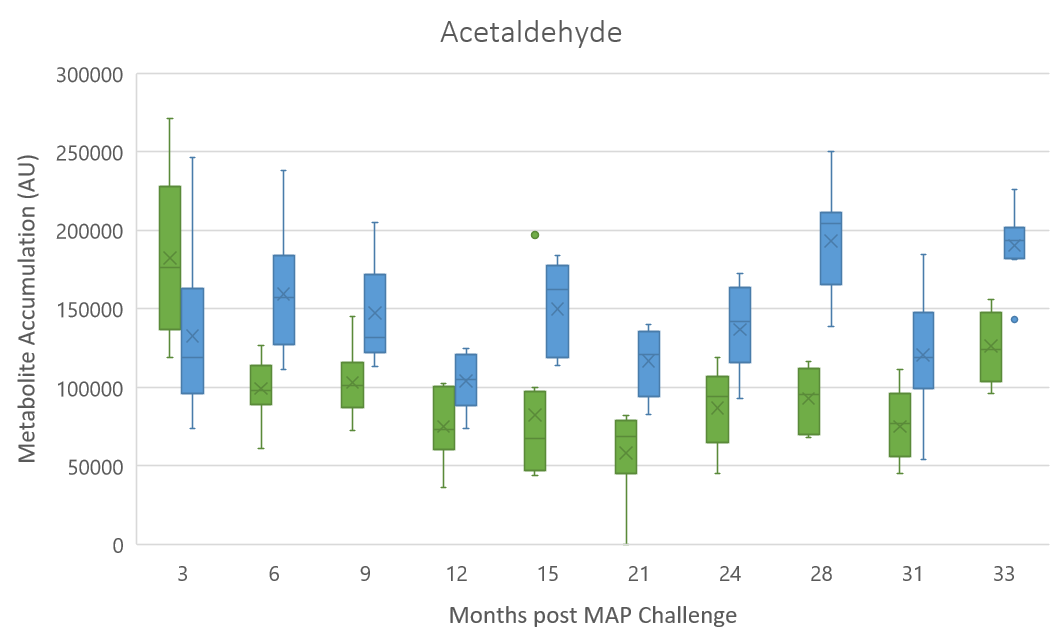

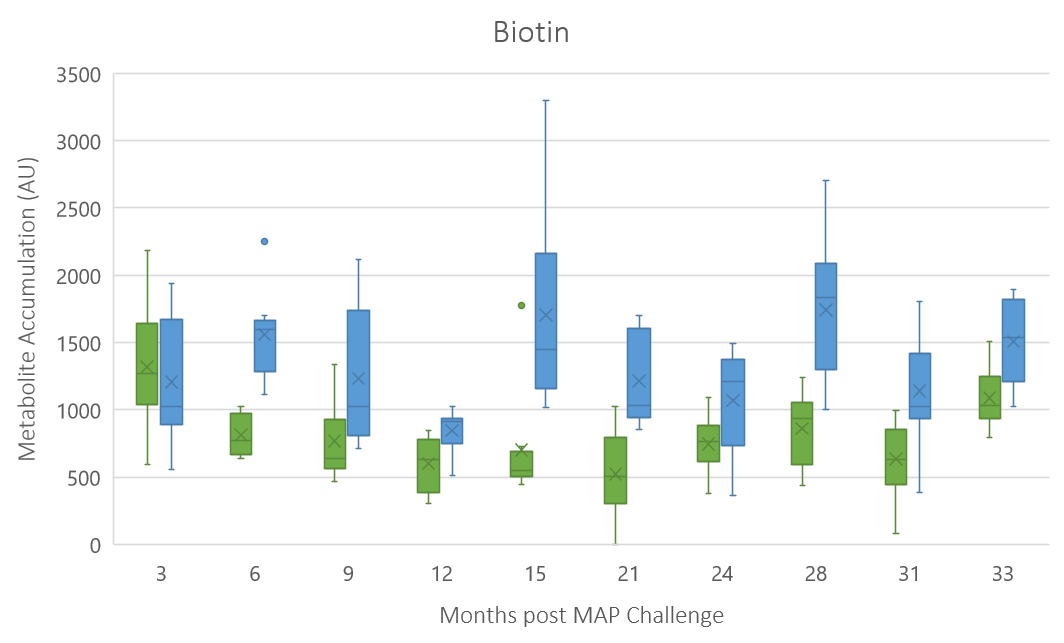

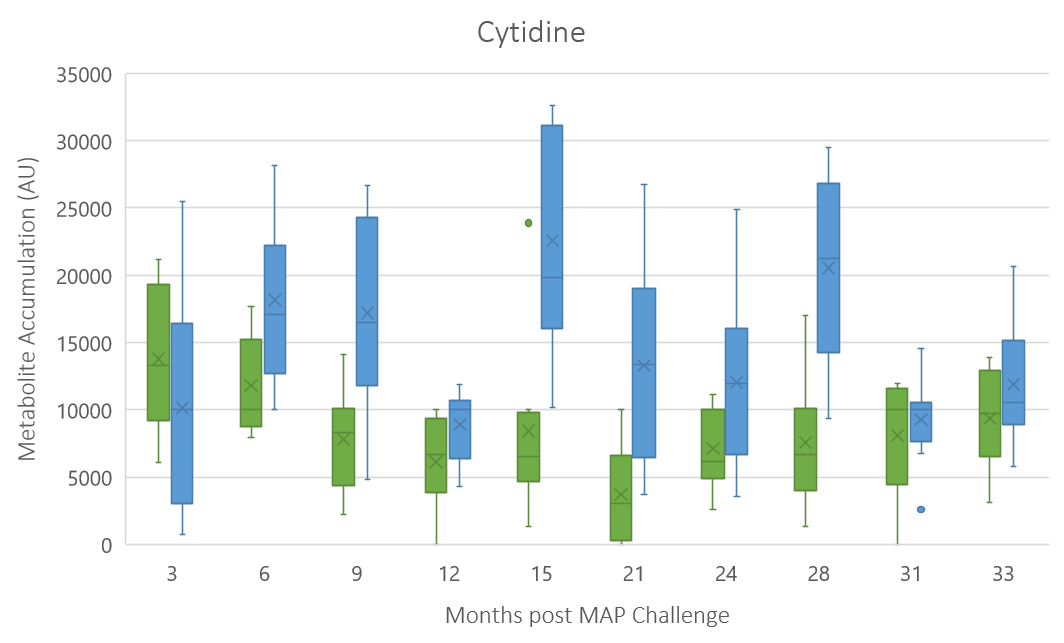

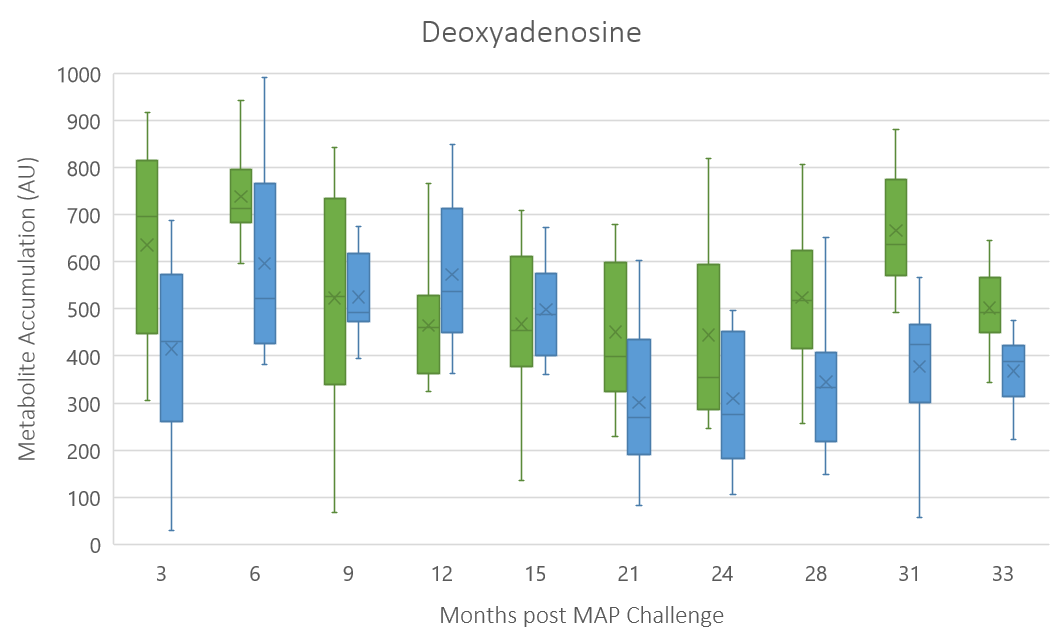

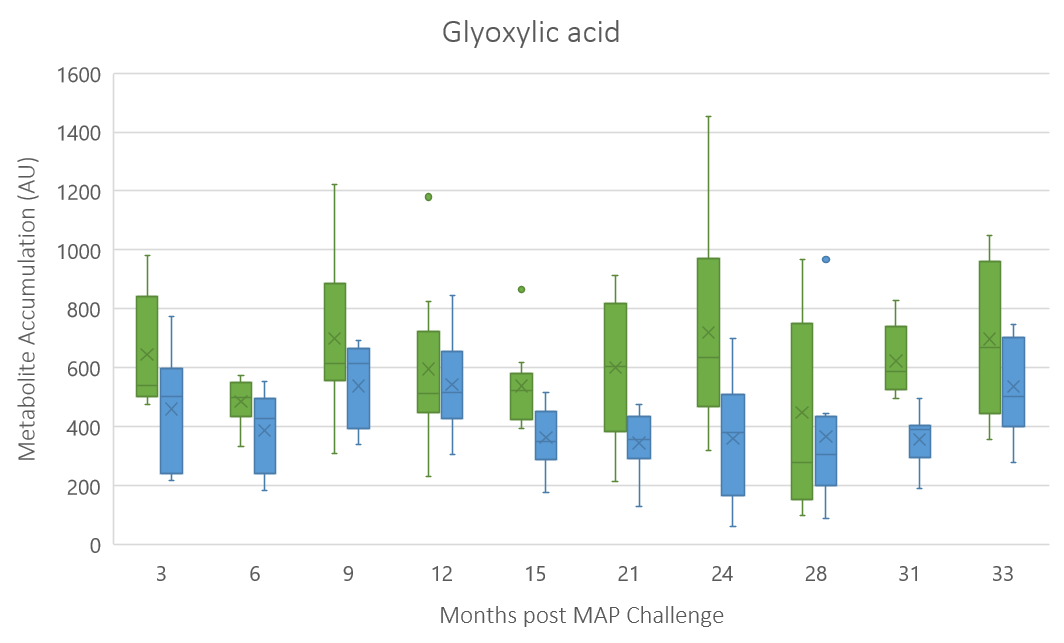

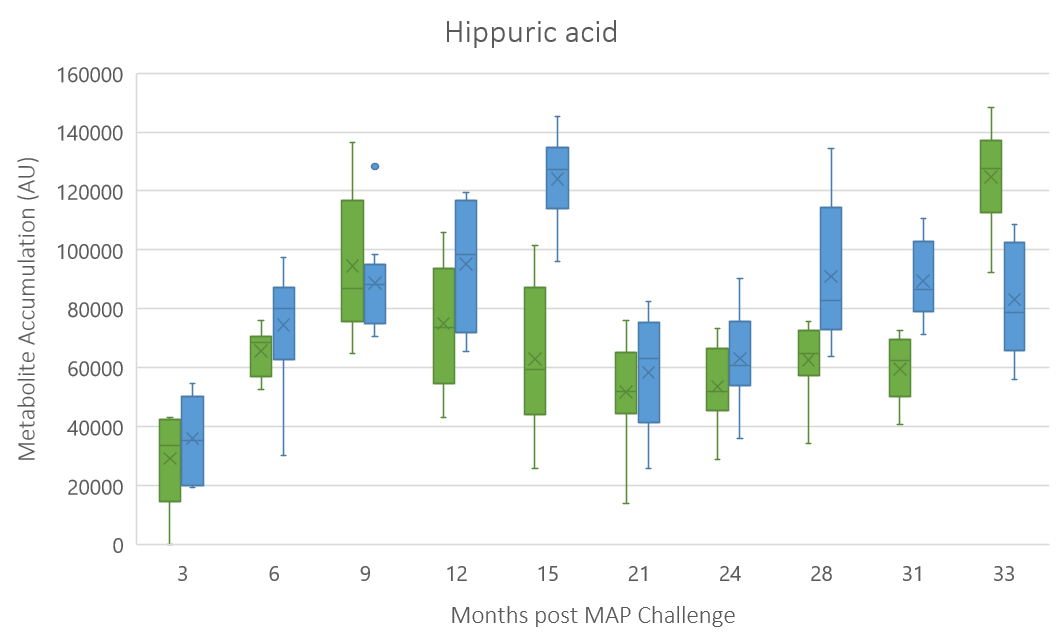

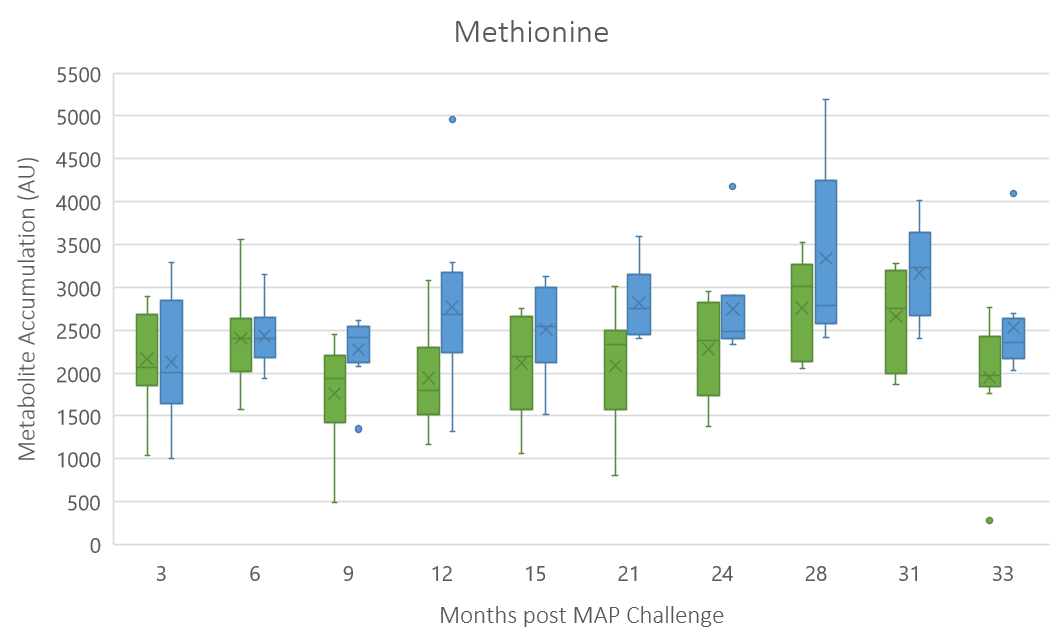

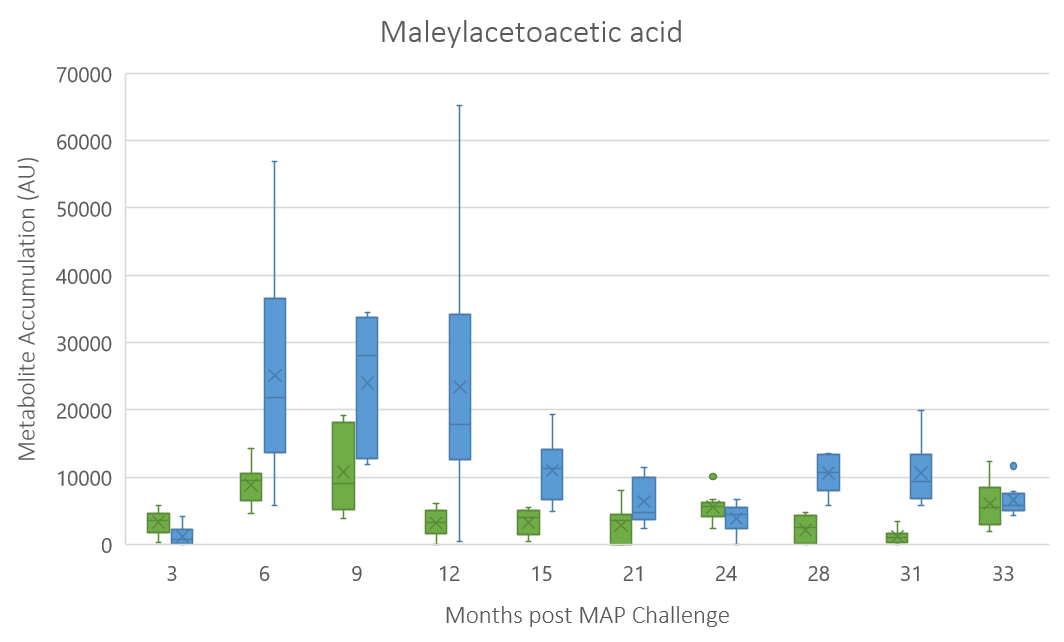

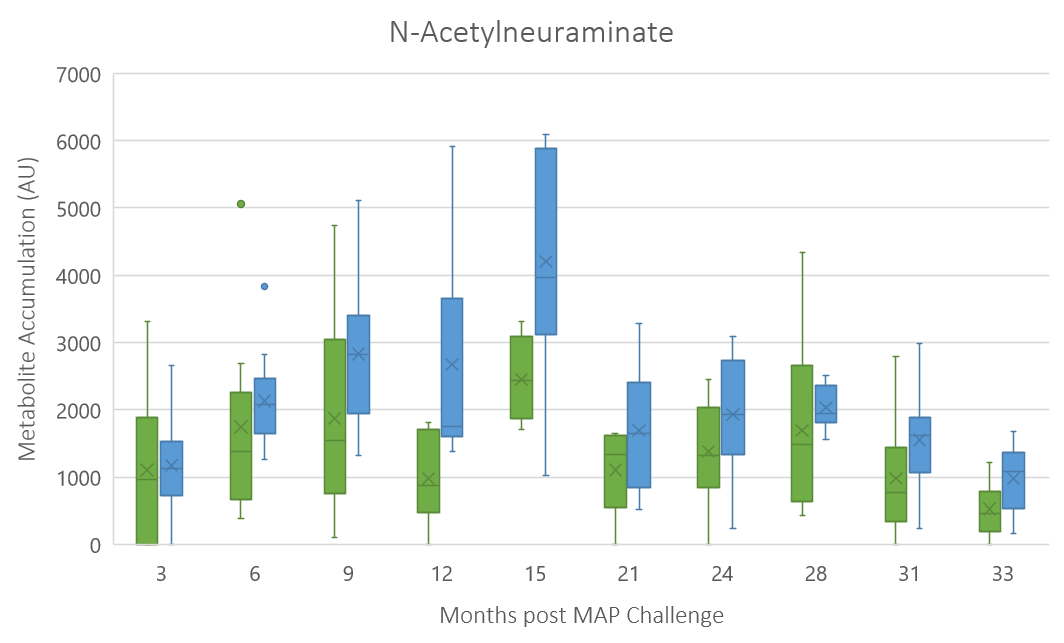

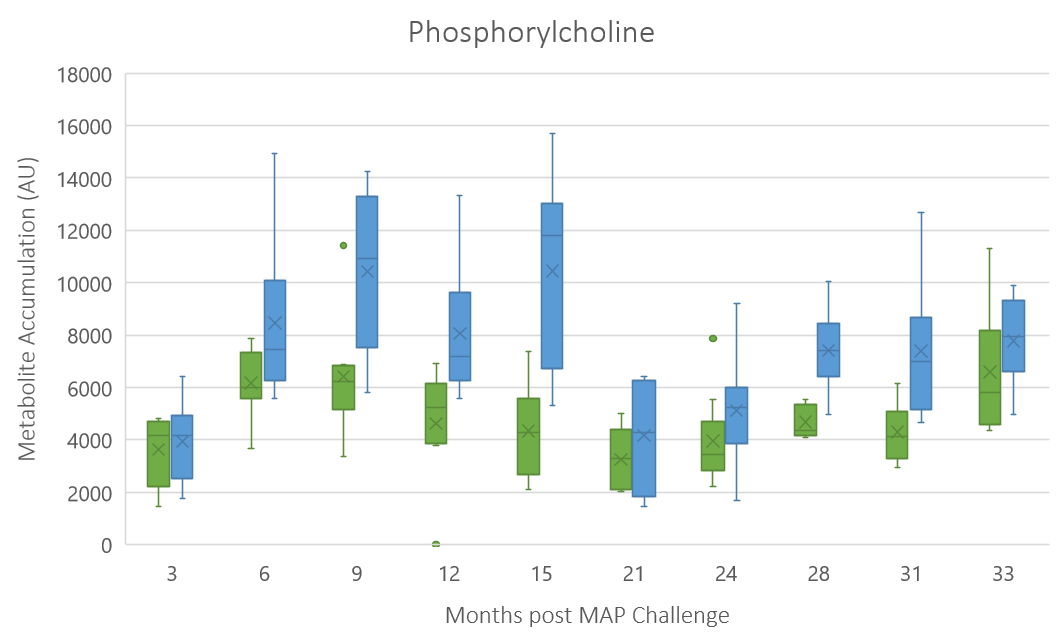

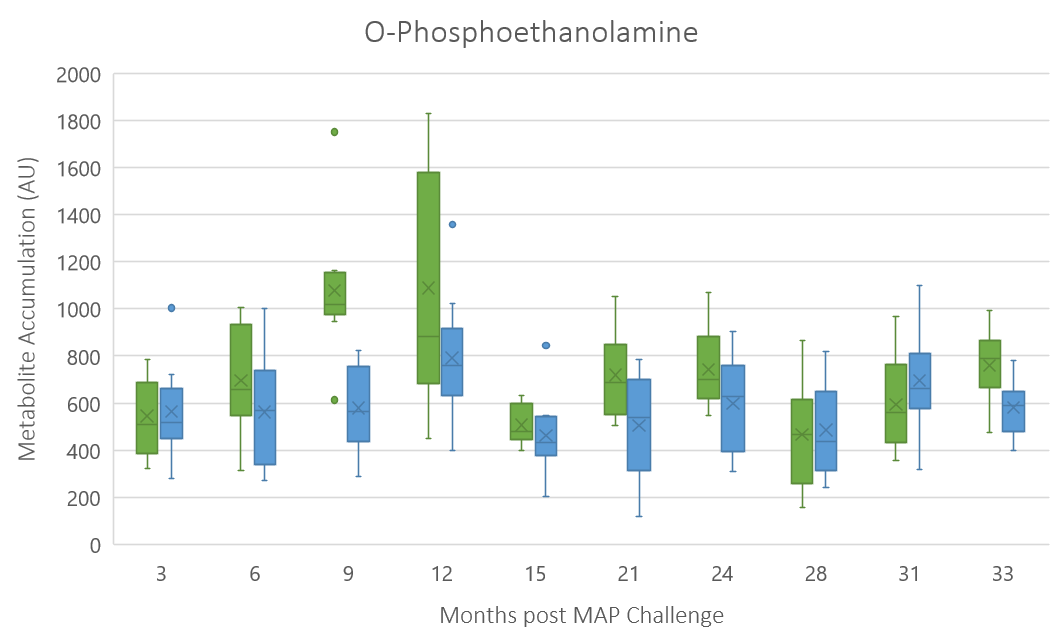

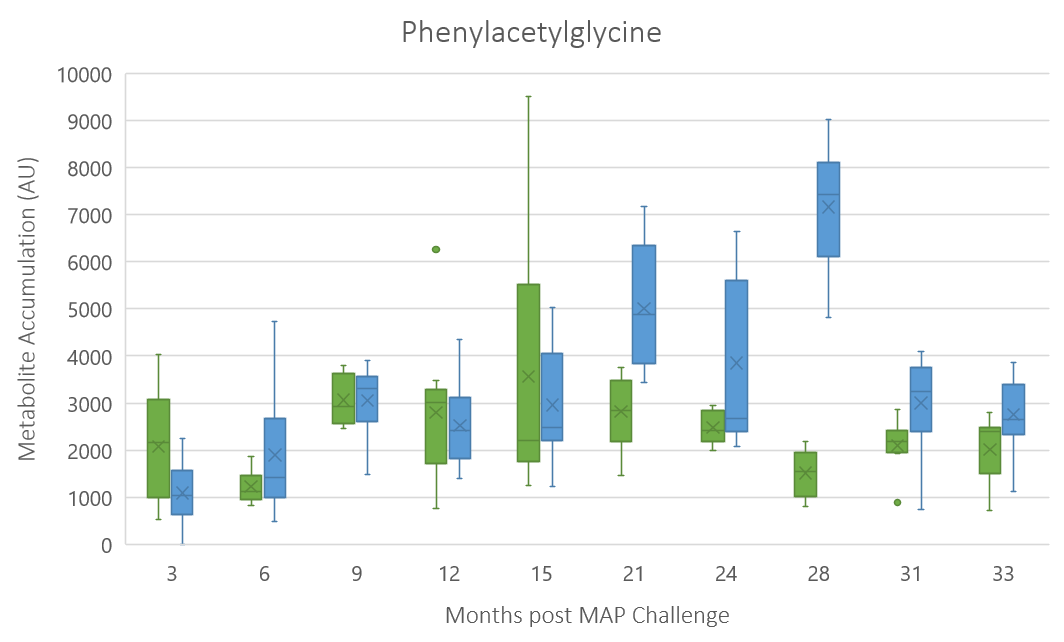

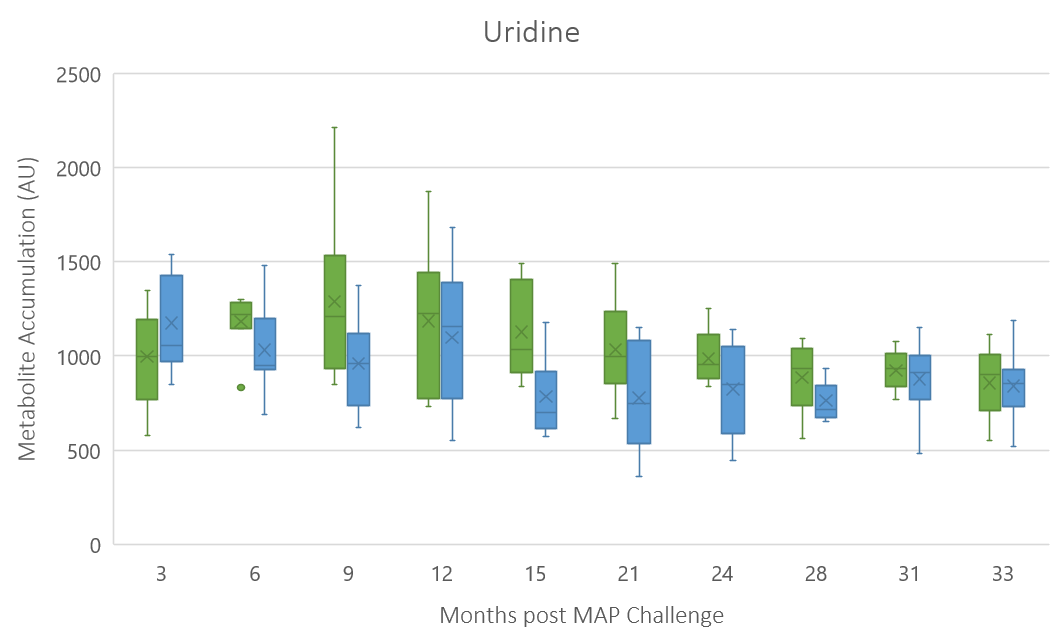


**Supplementary Fig 3** Box and whisker plots of metabolites between 3- and 33-months post MAP challenge in the negative ionisation mode *m/z*. Blue boxplots = MAP challenged cattle, green boxplots = control cattle.


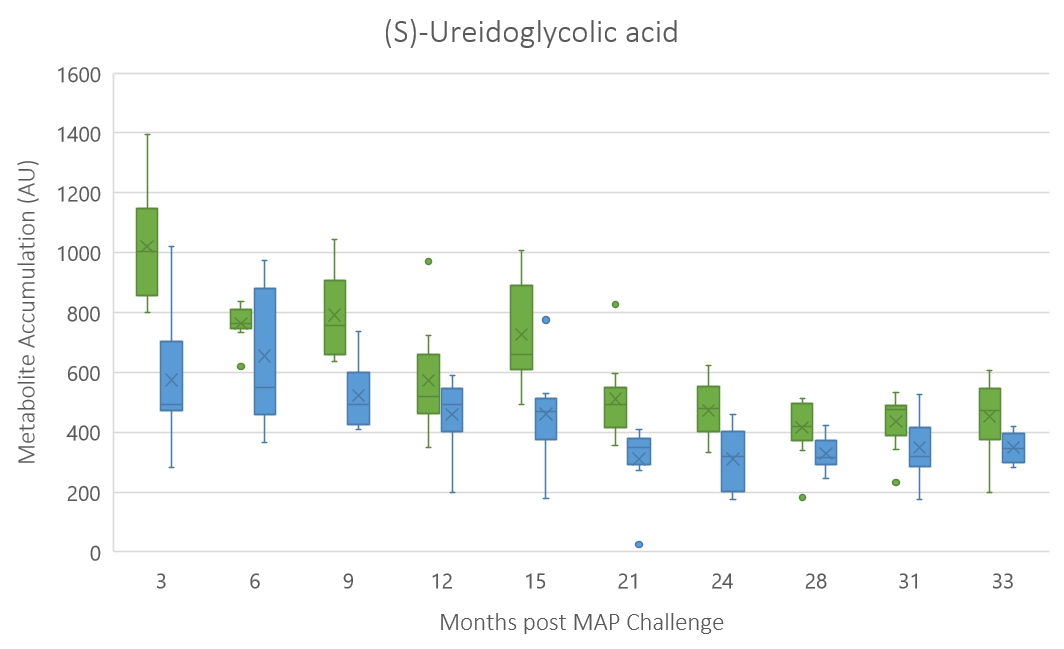

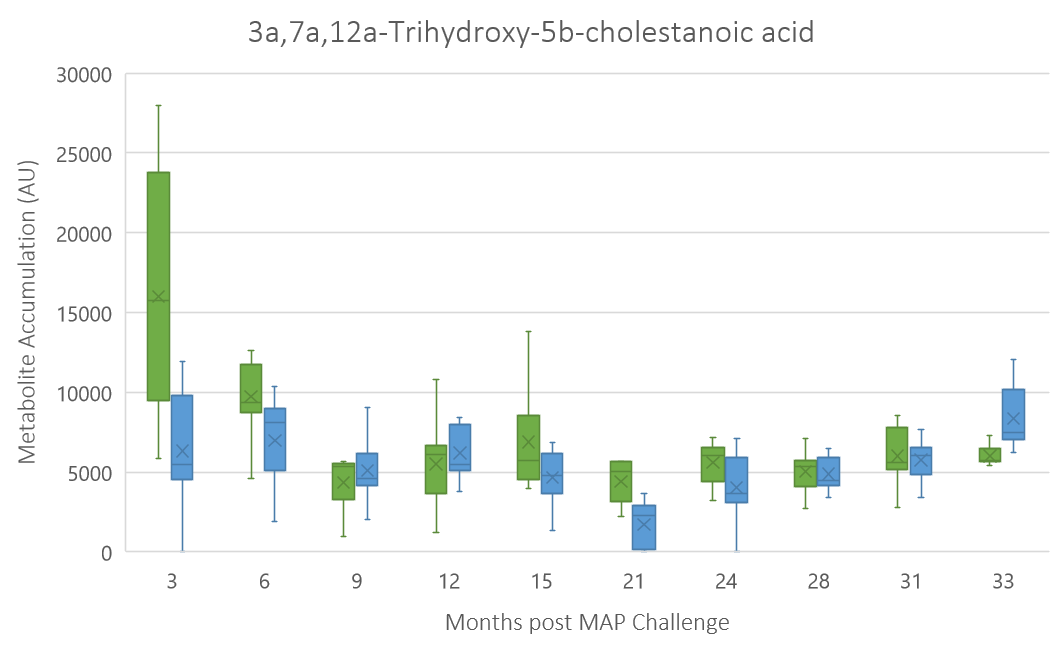

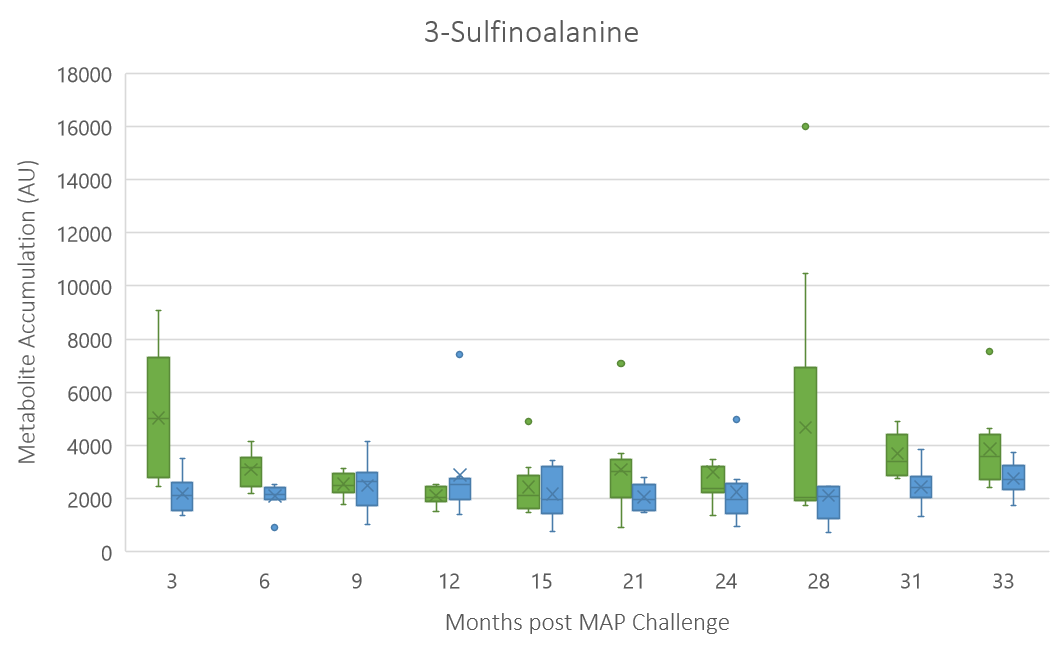

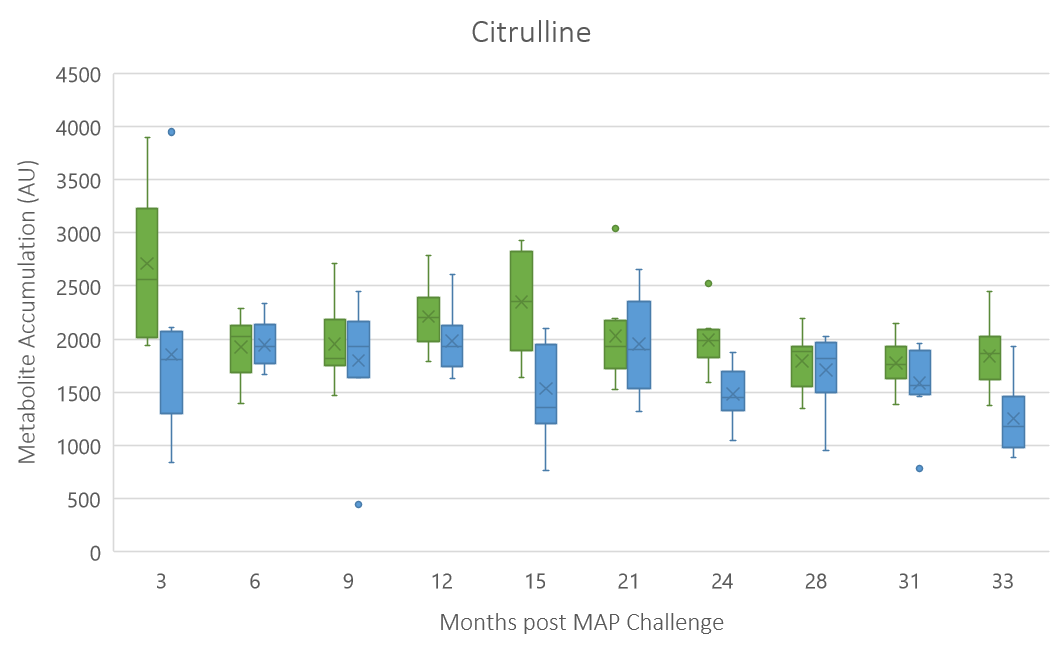

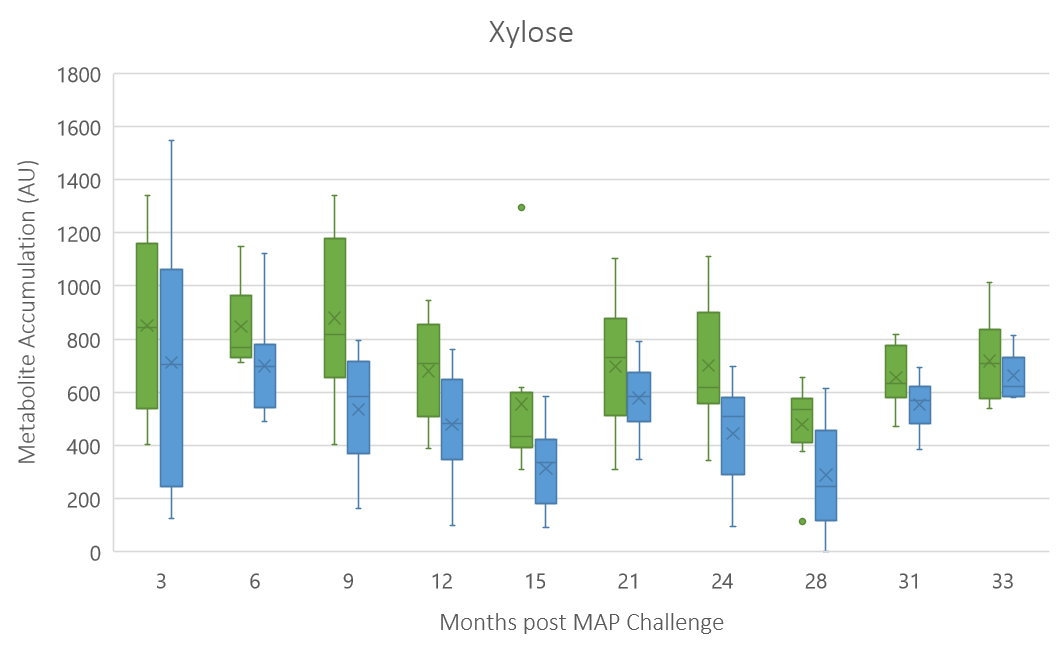

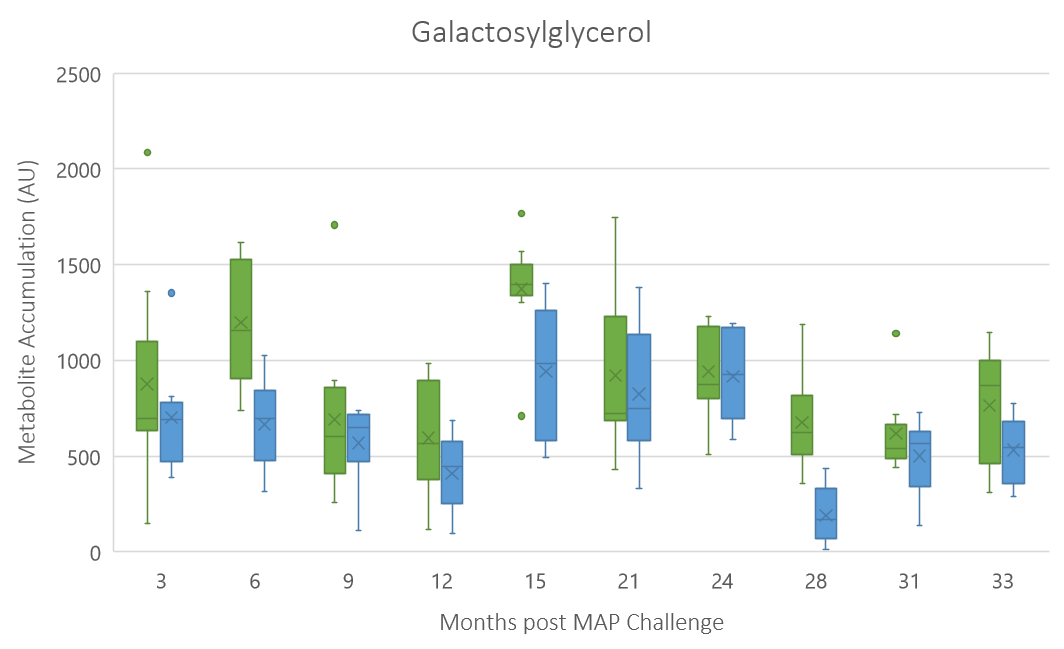

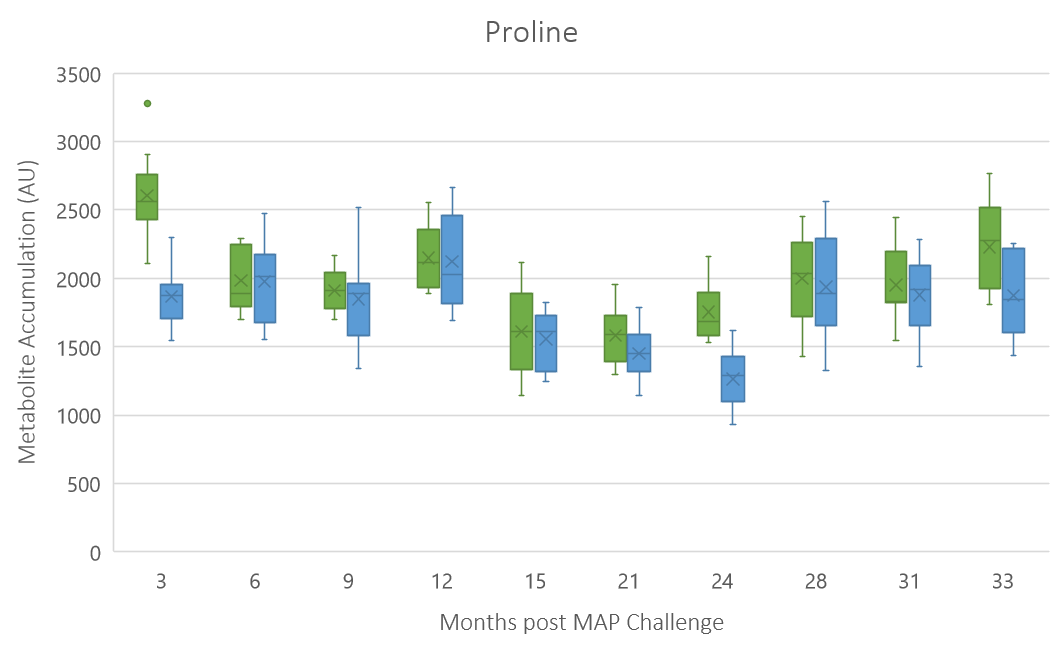

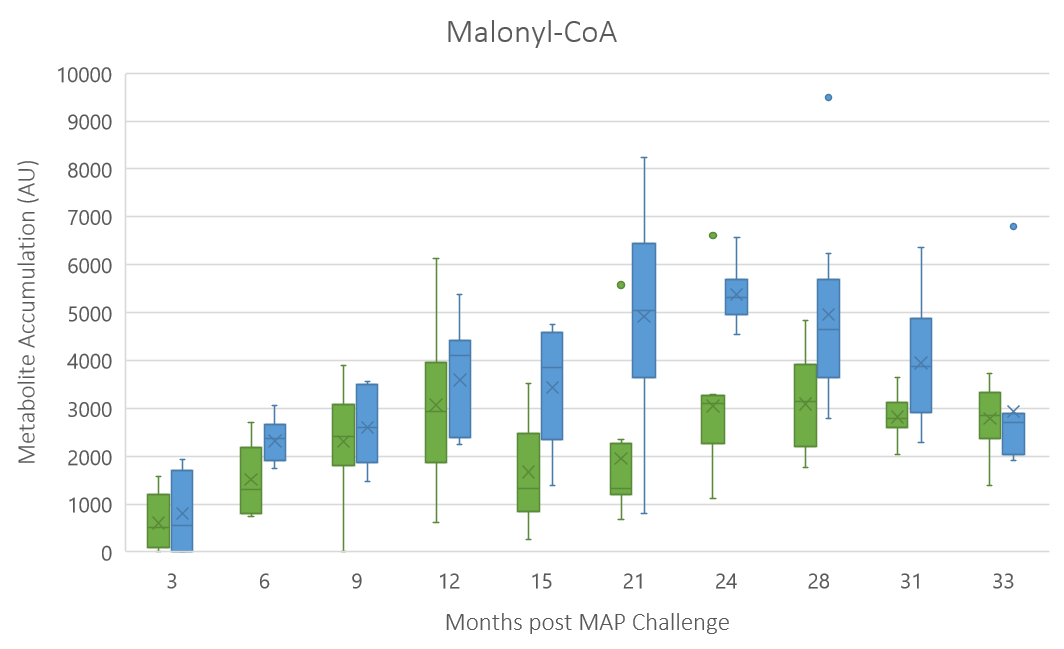


**Supplementary Fig 4** Box and whisker plots of metabolites between 3- and 33-months post MAP challenge in the positive ionisation mode *m/z*. Blue boxplots = MAP challenged cattle, green boxplots = control cattle.


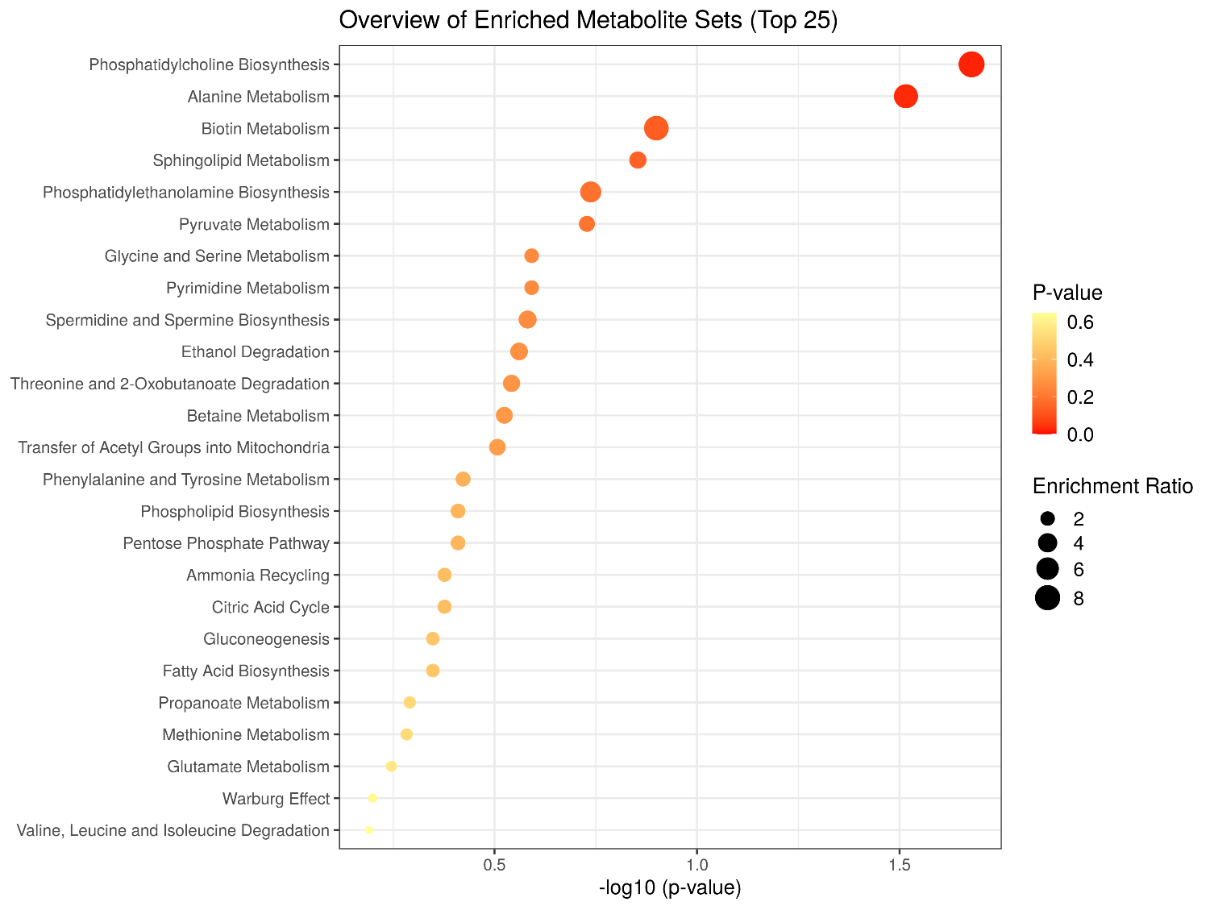


**Supplementary Fig 5** Significantly enriched pathways in MAP challenged cattle in the negative ionisation mode *m/z*.


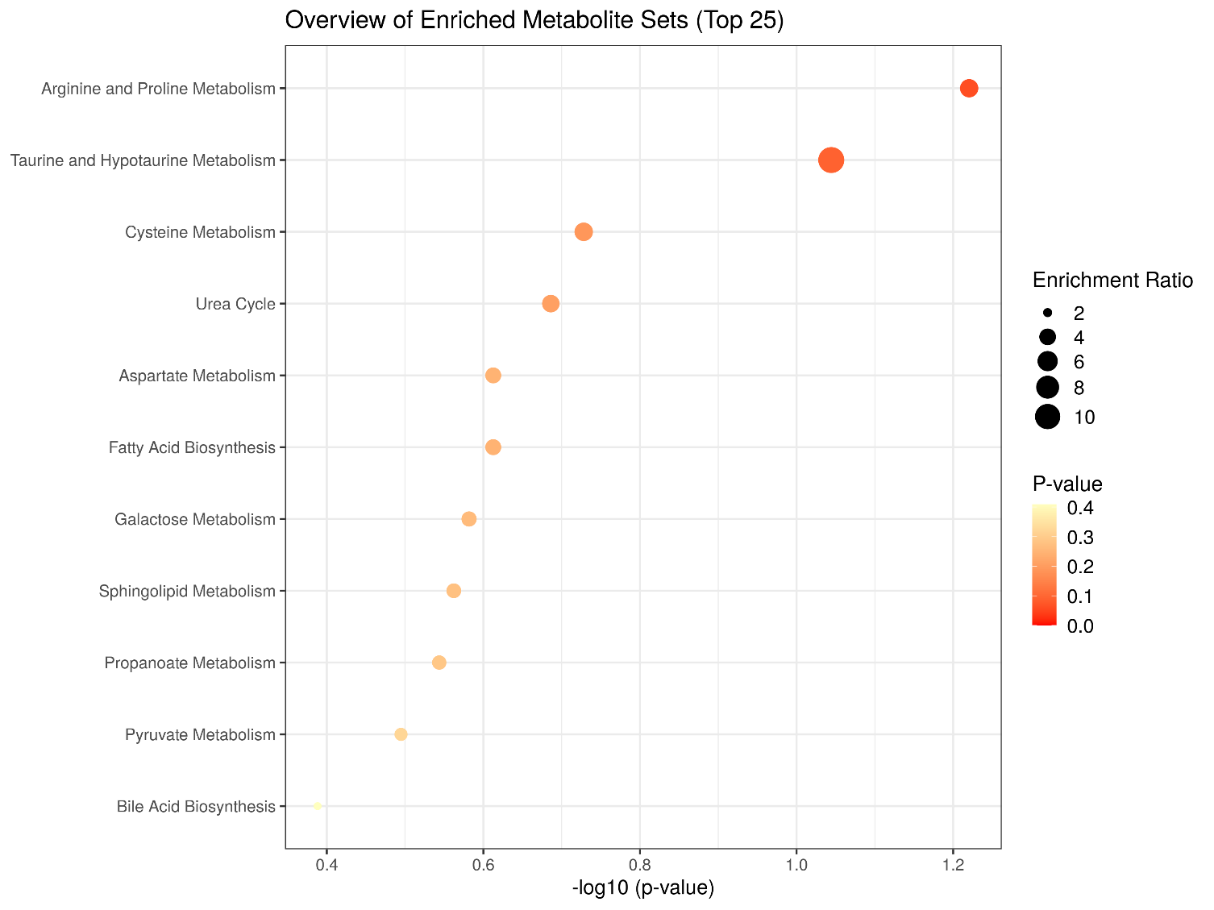


**Supplementary Fig 6** Significantly enriched pathways in MAP challenged cattle in the positive ionisation mode *m/z*.


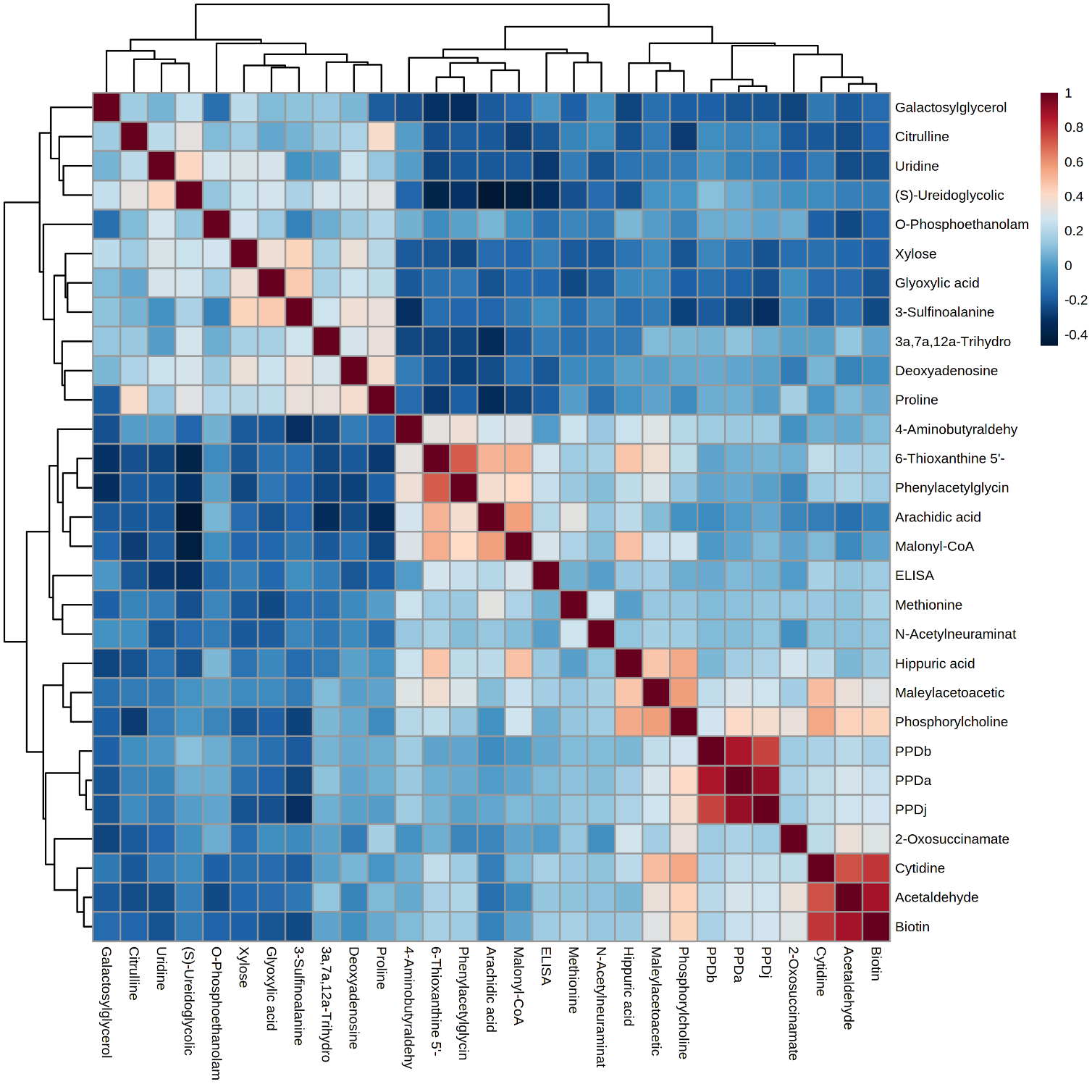


**Supplementary Fig 7** A heatmap of the Pearson’s correlation coefficients produced by comparing metabolites significantly affected by MAP challenge, PPDA, PPDB, PDDJ and ELISA between 3- and 33-months post MAP challenge. Positive correlations are shown in red, negative correlations are shown in blue.


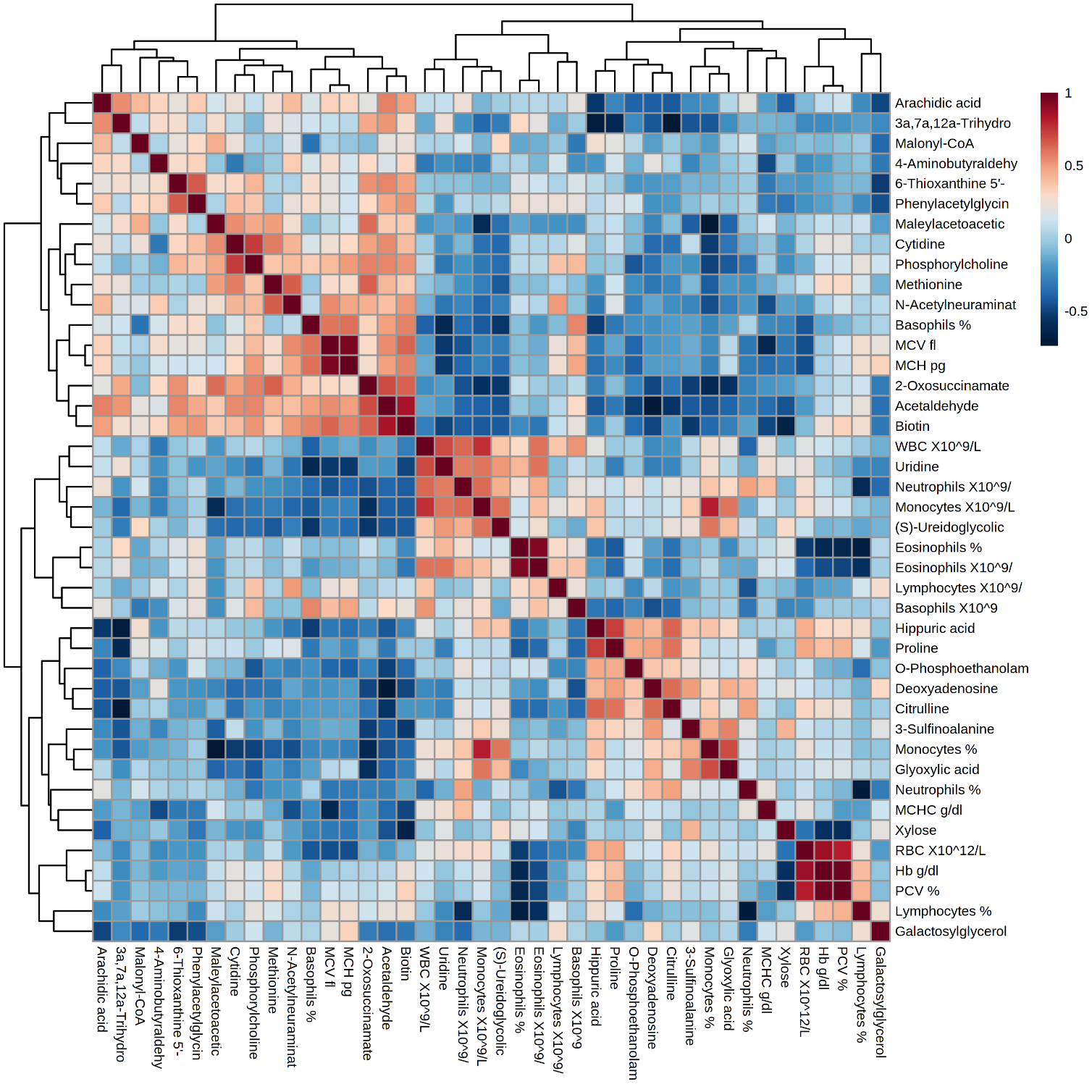


**Supplementary Fig 8** Pearson’s correlation coefficients between metabolites and hematology parameters 33-months post MAP challenge. Positive correlations are shown in red, negative correlations are shown in blue.


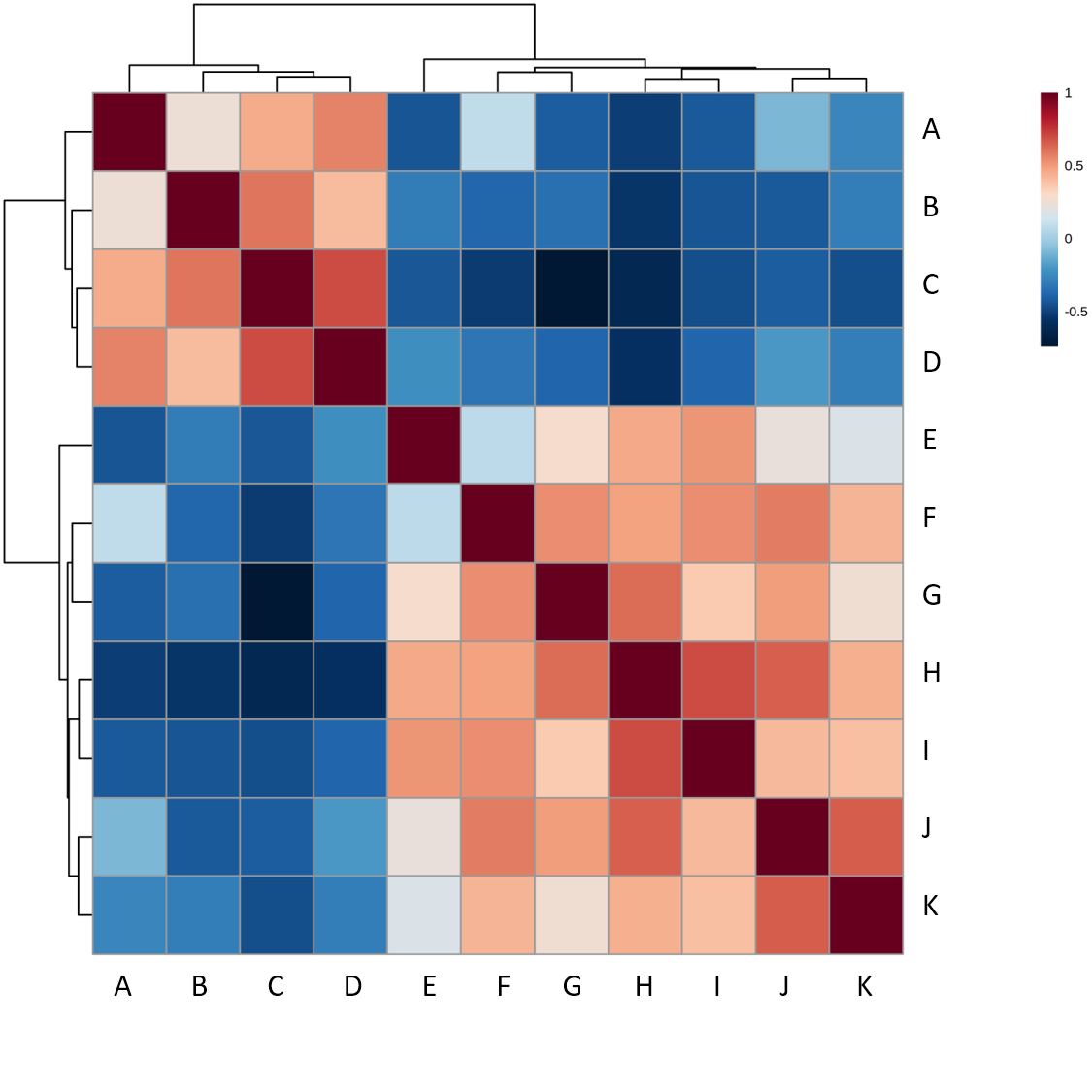


**Supplementary Fig 9** Pearson’s correlation coefficients between metabolites significantly affected by MAP challenge 33-months post MAP challenge, whereby -0.4 < correlation co-efficient > 0.4. A = 3-sulfinoalanine, B = (S)-Ureidoglycolic acid, C = monocyte (%), D = glyoxylic acid, E = 3a,7a,12a-trihydroxy-5b-cholestanoic acid, F = cytidine, G = maleylacetoacetic acid, H = 2-oxosuccinamate, I = acetaldehyde, J = methionine and K = N-acetylneuraminate Positive correlations are shown in red, negative correlations are shown in blue.


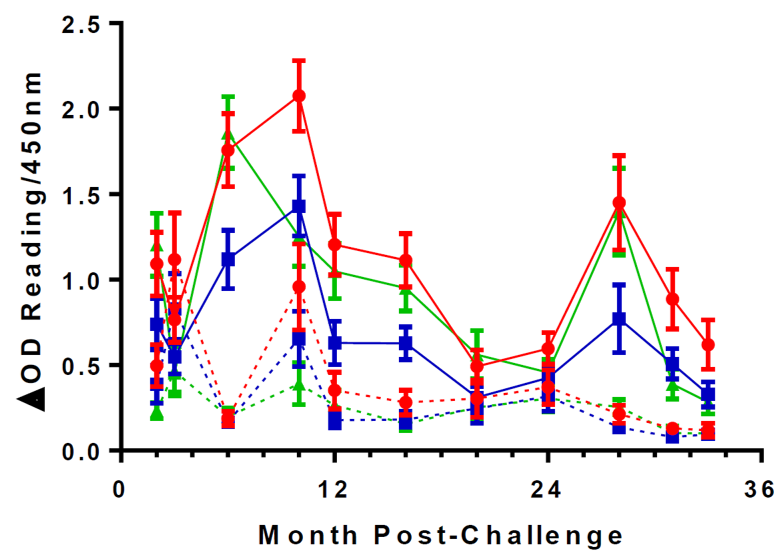


**Supplementary Fig 10** Mean± SEM of Bovigam® IGRA results of challenged (solid lines) and control (dashed lines) cattle in response to stimulation with PPDa (**O**), PPDb (**O**) and PPDj (**O**) over the course of the experimental challenge.

| **Supplementary Table 1.** Haematology results for MAP challenged and control cattle, 33-months post challenge. | | | | | | | | | | | | | | | | | | |  |
| --- | --- | --- | --- | --- | --- | --- | --- | --- | --- | --- | --- | --- | --- | --- | --- | --- | --- | --- | --- |
| **Group** | **ID** | **Hb g/dl** | **PCV %** | **RBC X10^12/L** | **WBC X10^9/L** | **MCV fl** | **MCHC g/dl** | **MCH pg** | **Lymphocytes%** | **Neutrophil %** | **Eosinophil %** | **Monocyte %** | **Basophil %** | **Lymphocyte X10^9/L** | **Neutrophil X10^9/L** | **Eosinophil X10^9/L** | **Monocyte X10^9/L** | **Basophil X10^9** | |
| MAP | 2149 | 11.8 | 33.7 | 6.04 | 4.1 | 55.8 | 35 | 19.5 | 54.8 | 31.6 | 9.4 | 2.5 | 1.4 | 2.22 | 1.28 | 0.38 | 0.1 | 0.06 | |
| MAP | 2155 | 13 | 35.5 | 7.74 | 5.4 | 45.9 | 36.6 | 16.8 | 53.8 | 32.9 | 9.4 | 3 | 0.9 | 2.89 | 1.77 | 0.5 | 0.16 | 0.05 | |
| MAP | 2176 | 13.3 | 37 | 7.42 | 5.1 | 49.9 | 35.9 | 17.9 | 73.4 | 19.9 | 3.7 | 2.1 | 0.8 | 3.71 | 1.01 | 0.19 | 0.11 | 0.04 | |
| MAP | 2194 | 13.1 | 36.8 | 7.14 | 5.4 | 51.5 | 35.6 | 18.3 | 69.9 | 21.3 | 5.2 | 2.6 | 0.7 | 3.77 | 1.15 | 0.28 | 0.14 | 0.04 | |
| MAP | 2201 | 10.7 | 29.5 | 6 | 7.4 | 49.2 | 36.3 | 17.8 | 58 | 20.8 | 17.7 | 2.5 | 0.9 | 4.28 | 1.54 | 1.31 | 0.18 | 0.07 | |
| MAP | 2212 | 14.6 | 41.1 | 7.87 | 8.7 | 52.2 | 35.5 | 18.6 | 75.3 | 17.9 | 3.6 | 2 | 0.9 | 6.57 | 1.56 | 0.31 | 0.17 | 0.08 | |
| MAP | 2387 | 11.4 | 32.5 | 6.43 | 5.2 | 50.5 | 35.1 | 17.7 | 60.4 | 32 | 4.1 | 2.5 | 0.8 | 3.12 | 1.65 | 0.21 | 0.13 | 0.04 | |
| MAP | 2390 | 12.7 | 36.9 | 6.95 | 7.1 | 53.1 | 34.4 | 18.3 | 66 | 21.4 | 6.9 | 4.6 | 0.7 | 4.67 | 1.51 | 0.49 | 0.33 | 0.05 | |
| MAP | 2402 | 13.8 | 39.2 | 7.74 | 8 | 50.6 | 35.2 | 17.8 | 70.3 | 18.5 | 5.3 | 4.4 | 0.8 | 5.6 | 1.47 | 0.42 | 0.35 | 0.06 | |
| Control | 2168 | 11.4 | 31.8 | 6.32 | 10.6 | 50.3 | 35.8 | 18 | 54.4 | 20.1 | 22.4 | 2.4 | 0.7 | 5.74 | 2.12 | 2.37 | 0.25 | 0.07 | |
| Control | 2218 | 11.6 | 32.1 | 7.05 | 5.8 | 45.5 | 36.1 | 16.5 | 65.1 | 22.2 | 9.4 | 2.8 | 0.5 | 3.76 | 1.28 | 0.54 | 0.16 | 0.03 | |
| Control | 2391 | 13.5 | 36.8 | 7.15 | 7.9 | 51.5 | 36.7 | 18.9 | 61.8 | 25.6 | 8 | 3.6 | 0.7 | 4.86 | 2.01 | 0.63 | 0.28 | 0.06 | |
| Control | 2395 | 13.7 | 37.7 | 8.29 | 8.4 | 45.5 | 36.3 | 16.5 | 50.8 | 35.5 | 9.8 | 3.3 | 0.5 | 4.26 | 2.98 | 0.82 | 0.28 | 0.04 | |
| Control | 2398 | 12.1 | 32.7 | 6.65 | 7.7 | 49.2 | 37 | 18.2 | 67.1 | 22.9 | 6 | 3.1 | 0.8 | 5.2 | 1.77 | 0.46 | 0.24 | 0.06 | |
| Control | 2405 | 12.6 | 34.7 | 7.22 | 6.8 | 48.1 | 36.3 | 17.5 | 63.2 | 25.6 | 6.6 | 3.7 | 0.7 | 4.32 | 1.75 | 0.45 | 0.25 | 0.05 | |
| Control | 2409 | 13.2 | 36.1 | 7.38 | 6.3 | 48.9 | 36.6 | 17.9 | 64.5 | 28 | 3 | 3.3 | 0.8 | 4.04 | 1.76 | 0.19 | 0.21 | 0.05 | |
| Control | 2425 | 11.6 | 32.1 | 6.94 | 7.7 | 46.3 | 36.1 | 16.7 | 61.3 | 25.6 | 7.5 | 4.7 | 0.8 | 4.7 | 1.96 | 0.58 | 0.36 | 0.06 | |
| Control | 2454 | 14.1 | 38.9 | 8.38 | 6.9 | 46.4 | 36.2 | 16.8 | 63.7 | 28.5 | 3.3 | 3.4 | 0.7 |  | 1.97 | 0.23 | 0.24 | 0.05 | |

| **Supplementary Table 2:** Area under the curve (AUC) assessments of the targeted metabolites | | | | | | | | | | | |  |
| --- | --- | --- | --- | --- | --- | --- | --- | --- | --- | --- | --- | --- |
| **Metabolite** | **Ionization Mode** | **Post MAP Challenge (months)** | | | | | | | | | |  |
|  |  | **3** | **6** | **10** | **12** | **16** | **20** | **24** | **28** | **31** | **33** | |
| 2-Oxosuccinamate | Negative | 0.809 | 0.642 | 0.938 | 0.735 | 0.827 | 0.599 | 0.840 | 0.969 | 0.895 | 0.864 | |
| 3a,7a,12a-Trihydroxy-5b-cholestanoic acid | Positive | 0.901 | 0.802 | 0.568 | 0.593 | 0.704 | 0.889 | 0.704 | 0.519 | 0.506 | 0.926 | |
| 3-Sulfinoalanine | Positive | 0.877 | 0.926 | 0.531 | 0.710 | 0.568 | 0.765 | 0.691 | 0.667 | 0.901 | 0.778 | |
| 4-Aminobutyraldehyde | Negative | 0.519 | 0.667 | 0.617 | 0.654 | 0.630 | 0.642 | 0.617 | 0.877 | 0.778 | 0.691 | |
| 6-Thioxanthine 5'-monophosphate | Negative | 0.630 | 0.951 | 0.778 | 0.679 | 0.549 | 1.000 | 1.000 | 1.000 | 0.889 | 0.802 | |
| Acetaldehyde | Negative | 0.790 | 0.938 | 0.914 | 0.852 | 0.889 | 1.000 | 0.914 | 1.000 | 0.877 | 0.975 | |
| Arachidic acid | Negative | 0.691 | 0.691 | 0.617 | 0.580 | 0.840 | 0.753 | 0.901 | 0.914 | 0.938 | 0.877 | |
| Biotin | Negative | 0.623 | 1.000 | 0.815 | 0.852 | 0.926 | 0.938 | 0.778 | 0.963 | 0.889 | 0.864 | |
| Citrulline | Positive | 0.827 | 0.531 | 0.537 | 0.716 | 0.864 | 0.556 | 0.951 | 0.975 | 0.654 | 0.895 | |
| Cytidine | Negative | 0.691 | 0.821 | 0.877 | 0.790 | 0.938 | 0.877 | 0.765 | 0.951 | 0.500 | 0.667 | |
| Deoxyadenosine | Negative | 0.790 | 0.765 | 0.556 | 0.716 | 0.568 | 0.765 | 0.679 | 0.840 | 0.975 | 0.901 | |
| Galactosylglycerol | Positive | 0.611 | 0.914 | 0.506 | 0.691 | 0.864 | 0.506 | 0.556 | 0.778 | 0.568 | 0.741 | |
| Glyoxylic acid | Negative | 0.722 | 0.741 | 0.593 | 0.506 | 0.883 | 0.802 | 0.809 | 0.531 | 1.000 | 0.667 | |
| Hippuric acid | Negative | 0.617 | 0.728 | 0.556 | 0.753 | 0.975 | 0.580 | 0.679 | 0.889 | 0.988 | 0.926 | |
| Maleylacetoacetic acid | Negative | 0.827 | 0.877 | 0.840 | 0.901 | 0.975 | 0.790 | 0.716 | 1.000 | 1.000 | 0.556 | |
| Malonyl-CoA | Positive | 0.568 | 0.802 | 0.568 | 0.605 | 0.864 | 0.840 | 0.889 | 0.852 | 0.815 | 0.605 | |
| Methionine | Negative | 0.531 | 0.500 | 0.815 | 0.790 | 0.704 | 0.846 | 0.654 | 0.667 | 0.667 | 0.765 | |
| N-Acetylneuraminate | Negative | 0.574 | 0.679 | 0.728 | 0.840 | 0.852 | 0.679 | 0.691 | 0.691 | 0.735 | 0.765 | |
| Phosphorylcholine | Negative | 0.568 | 0.778 | 0.877 | 0.901 | 0.951 | 0.605 | 0.704 | 0.963 | 0.926 | 0.741 | |
| O-Phosphoethanolamine | Negative | 0.506 | 0.654 | 0.963 | 0.679 | 0.679 | 0.765 | 0.691 | 0.519 | 0.673 | 0.846 | |
| Phenylacetylglycine | Negative | 0.753 | 0.642 | 0.512 | 0.543 | 0.568 | 0.975 | 0.704 | 1.000 | 0.840 | 0.802 | |
| Proline | Positive | 0.988 | 0.506 | 0.605 | 0.556 | 0.568 | 0.667 | 0.963 | 0.580 | 0.519 | 0.815 | |
| S-Ureidoglycolic acid | Positive | 0.938 | 0.605 | 0.963 | 0.654 | 0.901 | 0.926 | 0.877 | 0.802 | 0.778 | 0.827 | |
| Uridine | Negative | 0.654 | 0.741 | 0.753 | 0.568 | 0.852 | 0.704 | 0.691 | 0.747 | 0.580 | 0.531 | |
| Xylose | Positive | 0.605 | 0.802 | 0.821 | 0.778 | 0.802 | 0.654 | 0.815 | 0.506 | 0.784 | 0.543 | |

| **Supplementary Table 3** The number of correlations between haematology parameters and metabolites significantly affected by MAP challenge 33-months post MAP challenge whereby -0.4 < correlation co-efficient > 0.4 | |
| --- | --- |
| **Haematology Parameters** | **Frequency** |
| Monocytes % | 11 |
| MCH pg | 6 |
| Monocytes X10^9^/L | 6 |
| Basophils % | 5 |
| MCV fl | 5 |
| Neutrophils X10^9^/L | 4 |
| MCHC g/dl | 3 |
| Basophils X10^9^/L | 2 |
| Eosinophils % | 2 |
| RBC X10^12^/L | 2 |
| PCV % | 2 |
| Eosinophils X10^9^/L | 1 |
| Hb g/dl | 1 |
| Lymphocytes X10^9^/L | 1 |
| Neutrophils % | 1 |
| WBC X10^9^/L | 1 |
| Lymphocytes % | 0 |
